# Vitamin D deficiency serves as a precursor to stunted growth and central adiposity in zebrafish

**DOI:** 10.1038/s41598-020-72622-2

**Published:** 2020-09-29

**Authors:** Megan M. Knuth, Debabrata Mahapatra, Dereje Jima, Debin Wan, Bruce D. Hammock, Mac Law, Seth W. Kullman

**Affiliations:** 1 Toxicology Program, Department of Biological Sciences, North Carolina State University, Campus Box 7633, Raleigh, NC 27695-7633 USA; 2 Comparative Biomedical Sciences, College of Veterinary Medicine, North Carolina State University, Raleigh, NC 27606 USA; 3 Bioinformatics Research Center, North Carolina State University, Raleigh, NC 27606 USA; 4 Center for Human Health and the Environment, North Carolina State University, Raleigh, NC 27606 USA; 5 Department of Entomology and Nematology and University of California Davis Comprehensive Cancer Center, University of California Davis, Davis, CA 95616 USA; 6 Department of Population Health and Pathobiology, College of Veterinary Medicine, North Carolina State University, Raleigh, NC 27606 USA

**Keywords:** Genetics, Molecular biology, Physiology, Diseases, Endocrinology

## Abstract

Emerging evidence demonstrates the importance of sufficient vitamin D (1α, 25-dihydroxyvitamin D3) levels during early life stage development with deficiencies associated with long-term effects into adulthood. While vitamin D has traditionally been associated with mineral ion homeostasis, accumulating evidence suggests non-calcemic roles for vitamin D including metabolic homeostasis. In this study, we examined the hypothesis that vitamin D deficiency (VDD) during early life stage development precedes metabolic disruption. Three dietary cohorts of zebrafish were placed on engineered diets including a standard laboratory control diet, a vitamin D null diet, and a vitamin D enriched diet. Zebrafish grown on a vitamin D null diet between 2–12 months post fertilization (mpf) exhibited diminished somatic growth and enhanced central adiposity associated with accumulation and enlargement of visceral and subcutaneous adipose depots indicative of both adipocyte hypertrophy and hyperplasia. VDD zebrafish exhibited elevated hepatic triglycerides, attenuated plasma free fatty acids and attenuated lipoprotein lipase activity consistent with hallmarks of dyslipidemia. VDD induced dysregulation of gene networks associated with growth hormone and insulin signaling, including induction of suppressor of cytokine signaling. These findings indicate that early developmental VDD impacts metabolic health by disrupting the balance between somatic growth and adipose accumulation.

## Introduction

Vitamin D deficiency (VDD) is a widespread nutrient deficiency characterized by serum 25,hydroxyvitamin D (25(OH)D) levels below 20 ng/mL^1,2^. Globally, VDD impacts roughly one billion individuals as a result of dietary depletion, insufficient sunlight exposure, or genetic variants^1^. In recent years, the vitamin D/VDR (vitamin D receptor) signaling axis has been implicated in metabolic control, where low systemic vitamin D levels are associated with obesity^3^. Similar to the increasing incidence of VDD, the occurrence of obesity has tripled since 1975 with nearly two billion obese individuals globally^4^. Both VDD and obesity have been recognized as pandemic, and while the etiology for VDD is well understood, the cause of obesity varies greatly from patient to patient with emerging evidence suggesting that VDD may play an important role^5,6^.

Over the past decade, evidence has linked VDD with low birth weight, small for gestational age, impaired neurodevelopment, autoimmune disease, type 1 and type 2 diabetes (T1D, T2D, respectively), and cardiometabolic dysfunctions including obesity and insulin resistance (IR), suggesting a role for vitamin D in the pathogenesis of metabolic disorders^6–13^. Evidence further suggests that elevated levels of vitamin D are associated with a decrease in relative risk for T1D, T2D, hyperglycemia, and IR^14–16^.

In human population studies there is increasing evidence of a physiological role of vitamin D in glucose and lipid metabolism within insulin-sensitive tissues expressing VDR^17^. In adipose tissue (AT), vitamin D is thought to regulate markers of adipogenesis, such as adiponectin, as well as adipocyte apoptosis^18^. While in the liver and muscle, vitamin D levels are inversely associated with the prevalence of non-alcoholic fatty liver disease and muscle weakness^19,20^. Studies also indicate that higher body mass indices (BMI’s) are coupled with lower vitamin D levels and a higher risk of developing IR and T2D^21,22^. One study involving women ages 16–22 years old, demonstrates a strong correlation between low vitamin D levels and reduced height (cm), increased weight (kg), and increased BMI (kg/m^2^)^23^. Interestingly, recent epidemiologic studies suggest that VDD may precede the onset of obesity and progression of IR and T2D^24^. This is most prevalent in studies examining maternal vitamin D status and birth outcomes. Several recent studies demonstrate a correlation between low maternal vitamin D levels during pregnancy and small gestational size followed by high BMI’s at one and three years of age^25^. These studies provide initial evidence for an inverse relationship between maternal vitamin D levels and adverse metabolic health outcomes in offspring including excess adiposity and insulin resistance; however, not all studies have demonstrated a definite relationship between VDD and cardiometabolic risk factors in offspring^6^.

Metabolic research utilizing zebrafish is still relatively new to the field; however, functional conservation of key pathways associated with energy homeostasis, such as lipid metabolism and adipogenesis, make the zebrafish model a novel tool for characterizing lipid development before, during, and after the onset of metabolic diseases^26^. Genetic zebrafish models disrupting the vitamin D signaling cascade support results found in 3T3-L1 cells, with the absence of vitamin D promoting pro-adipogenic phenotypes. *Cyp2r1*^–/–^ zebrafish are obese by 100 days post-fertilization (dpf), with elevated levels of visceral and subcutaneous fat, and significantly stunted growth compared to wildtype (WT) controls^27^. Similarly, zebrafish mutants with disrupted GH signaling display a comparable obese phenotype with hypertrophy of AT accompanied by stunted growth as early as 12 dpf, and overexpression of downstream signaling molecule *akt1* results in zebrafish with elevated BMIs, AT hyperplasia, and impaired glucose clearance by 5 mpf^28–30^. These models indicate a potential link between disruption in vitamin D and GH signaling, and obesity.

In this study, we sought to address the hypothesis that VDD during early life stage development serves as a precursor to metabolic disruption. Three dietary cohorts of zebrafish were placed on engineered diets including a standard laboratory control diet (LD; 1.4 iu/g vitamin D), a vitamin D null diet (0 iu/g vitamin D), and a vitamin D enriched diet (400,000 iu/g vitamin D). Zebrafish grown on a vitamin D null diet between 2–12 mpf exhibited significantly stunted somatic growth and central adiposity. These findings indicate that early developmental VDD impacts metabolic health by disrupting the balance between somatic growth and adipose accumulation and suggests a unique linkage between vitamin D, VDD, and metabolic homeostasis.

## Results

### Confirming a state of VDD

**Figure 1:**
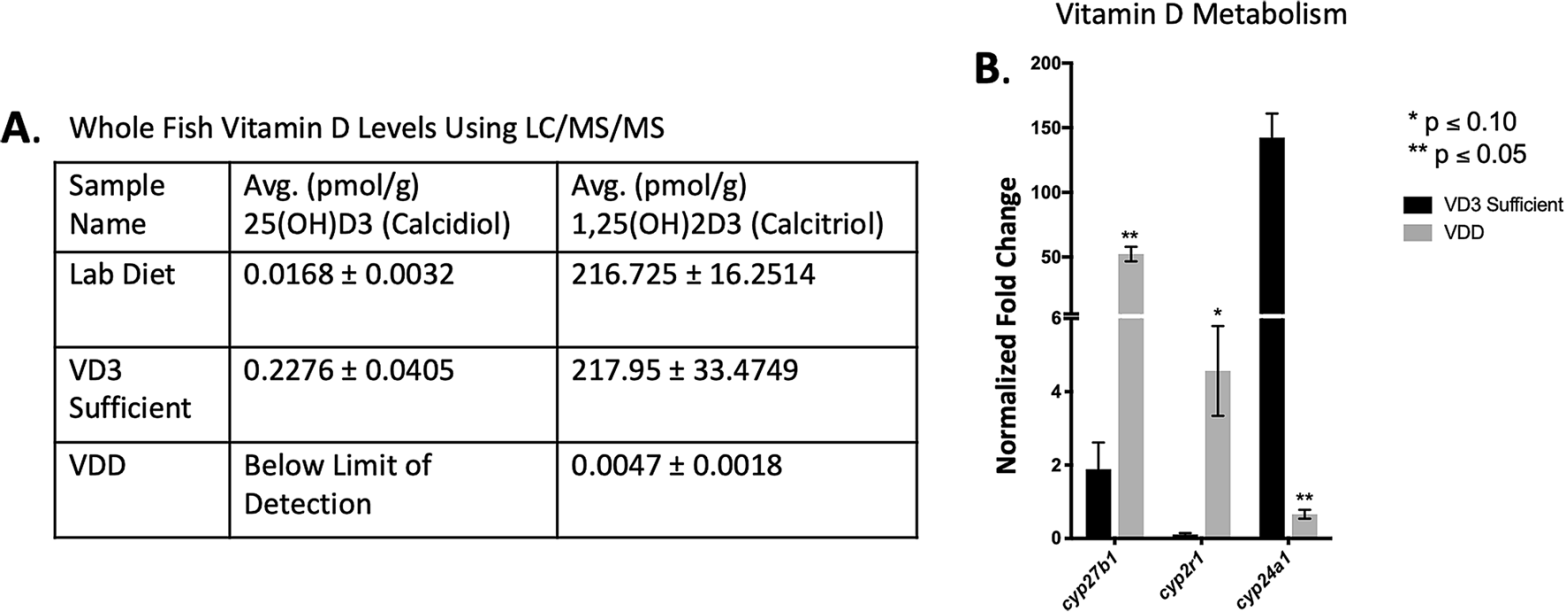
VDD in male 6 mpf zebrafish fed a vitamin D null diet. (**A**) VDD fish had lower whole body levels of both 25(OH)D3 and 1,25(OH)2D3 than lab diet and VD3 sufficient fish. The limit of quanititation (LOQ) was 8 pmol/g and the limit of detection (LOD) was 3 pmol/g. Data are represented as mean ± SD. (**B**) VDD livers expressed significantly more *cyp27b1* and *cyp2r1*, and significantly less *cy24a1*, than vitamin D sufficient livers. Data are represented as mean ± SE. *See also Supplementary Table 2.

To assess vitamin D sufficiency/deficiency whole fish vitamin D levels were measured using LC/MS/MS. As demonstrated in Fig. 1A, VDD zebrafish contained attenuated concentrations of both 1,25(OH)2D3 (0.0047 ± 0.0018 VDD versus 217.95 ± 33.4748 VD3 Sufficient) and the primary vitamin D metabolite 25(OH)D3 (below limit of detection VDD versus 0.2276 ± 0.0405 VD3 Sufficient). To confirm VDD at a molecular level we targeted select genes associated with vitamin D biosynthesis/metabolism known to undergo autoregulation. In this analysis it was found that VDD livers expressed significantly more *cyp27b1* and *cyp2r1*, and significantly less *cy24a1*, than vitamin D sufficient livers (Fig. 1B). Subsequently, whole fish bone density and whole fish calcium levels were examined using microCT and ICP MS, respectively (Supplementary Tables 2–3). We found that compared to the vitamin D sufficient cohort, VDD zebrafish had significantly attenuated total volume (78.33 ± 5.50 mm^3^ VDD versus 209.0 ± 50.17 mm^3^ VD3 Sufficient), bone volume (2.01 ± 0.43 mm^3^ VDD versus 7.12 ± 2.70 mm^3^ VD3 Sufficient), whole fish density (− 41.39 ± 3.04HU VDD versus − 30.15 ± 2.55HU VD3 Sufficient), bone density (427.08 ± 27.12HU VDD versus 560.18 ± 14.13HU VD3 Sufficient), and bone surface area (107.11 ± 16.34 mm^3^ VDD versus 317.56 ± 93.66 mm^3^ VD3 Sufficient) (Supplementary Table 2), and less whole fish calcium (3060.24 ± 442.05ug/g VDD versus 4656.1194 ± 499.50ug/g VD3 Sufficient) at 6 mpf (Supplementary Table 3). Taken together, these results confirm a state of VDD in the vitamin D null dietary group.

### Stunted growth and central adiposity are prominent phenotypes of VDD in zebrafish

**Figure 2:**
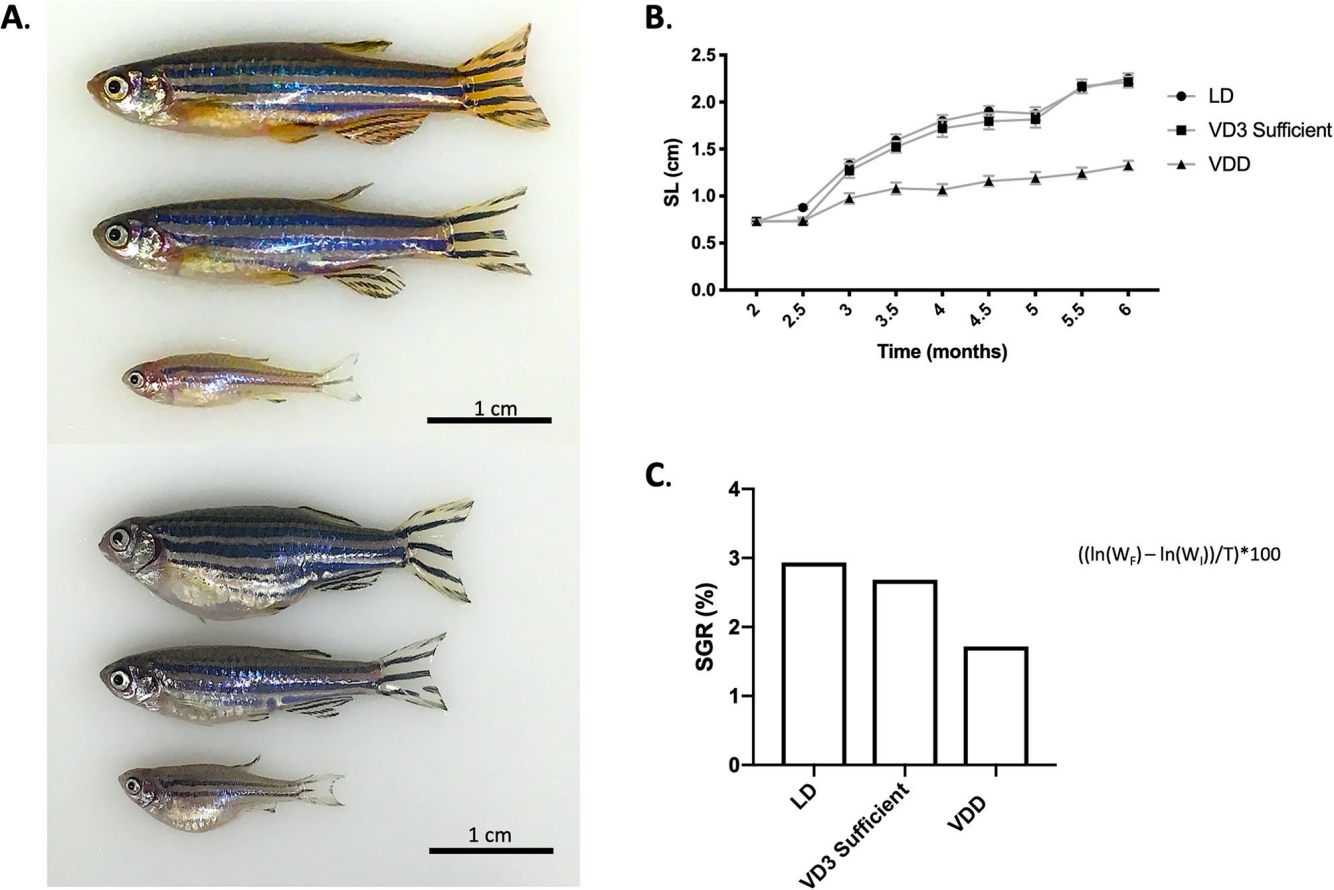
Stunted growth observed in the VDD zebrafish 6 mpf. (**A**) Both male and female VDD zebrafish exhibited stunted growth 6 mpf. Descending order: MALE (lab diet, VD3 sufficient, VDD), FEMALE (lab diet, VD3 sufficient, VDD). (**B**) Growth rate was taken biweekly starting at 2 mpf and ending at 6 mpf (2 mpf, 2.5 mpf, 3 mpf, etc.). At 6 mpf, the average SL (cm) for VDD, VD3 sufficient, and LD fish was 1.33 ± 0.0 cm, 2.21 ± 0.06 cm, and 2.25 ± -0.06 cm, respectively. Data are represented as mean ± SEM. (**C**) The SGR (%) from 2–6 mpf for VDD, VD3 sufficient, and LD fish was 1.72%, 2.69%, and 2.93%, respectively. Data are represented as mean ± SEM. All measures are representative of a mixed gender population. *See also Supplementary Figs. 1 and 2.

Both male and female VDD zebrafish exhibited stunted growth compared to zebrafish fed a vitamin D sufficient diet by 6 mpf (Fig. 2). Stunted growth was determined through biweekly measurements of growth rate between 2 and 6 mpf (Fig. 2B). At 6 mpf, the average SL (cm) for VDD, VD3 sufficient, and LD zebrafish was 1.33 ± 0.0 cm, 2.21 ± 0.06 cm, and 2.25 ± 0.06 cm, respectively. Standard Growth Rate (%) between 2–6 mpf exhibited a similar trend with VDD, VD3 sufficient, and LD zebrafish, 1.72%, 2.69%, and 2.93%, respectively (Fig. 1B). In addition to exhibiting a shorter SL and lower SGR, both male and female VDD zebrafish exhibited decreased body weight by 6 mpf (Supplementary Fig. 1A). At 6 mpf, the average body weight (g) for VDD, VD3 sufficient, and LD zebrafish was 0.0525 ± 0.007 g, 0.1761 ± 0.015 g, and 0.2403 ± 0.0230 g, respectively (Supplementary Fig. 1A), with a BWI (g) at 6 mpf of 0.0464 g, 0.1700 g, and 0.2342 g, respectively (Supplementary Fig. 1B).

To determine if re-introducing vitamin D into the vitamin D null diet could promote growth in VDD zebrafish, 6 mpf VDD animals were re-established on a vitamin D sufficient diet and monitored biweekly for SL (cm) and weight (g) through 11 mpf. Within two weeks (6.5 mpf) of supplementation, VDD + VD3 zebrafish exhibited greater SL and weight than zebrafish maintained on the VDD diet (1.56 ± 0.06 cm; 0.07 ± 0.01 g VDD + VD3 versus 1.25 ± 0.05 cm; 0.04 ± 0.01 g VDD) (Supplementary Fig. 2). This trend continued through the 11 mpf timepoint. To determine if hyperphagia contributed to the central adiposity observed in the VDD zebrafish, feeding rate was taken every month starting at 2 mpf and ending at 6mpf (Supplementary Fig. 1C). The feeding rate (%) at 6 mpf for VDD, VD3 sufficient, and LD zebrafish was 4.11 ± 0.0006%, 4.68 ± 0.0030%, and 1.62 ± 0.0020%, respectively, demonstrating that hyperphagia did not contribute to the observed central adiposity. Taken together, these results confirm stunted growth in the VDD zebrafish staring as early as 3 mpf.

**Figure 3:**
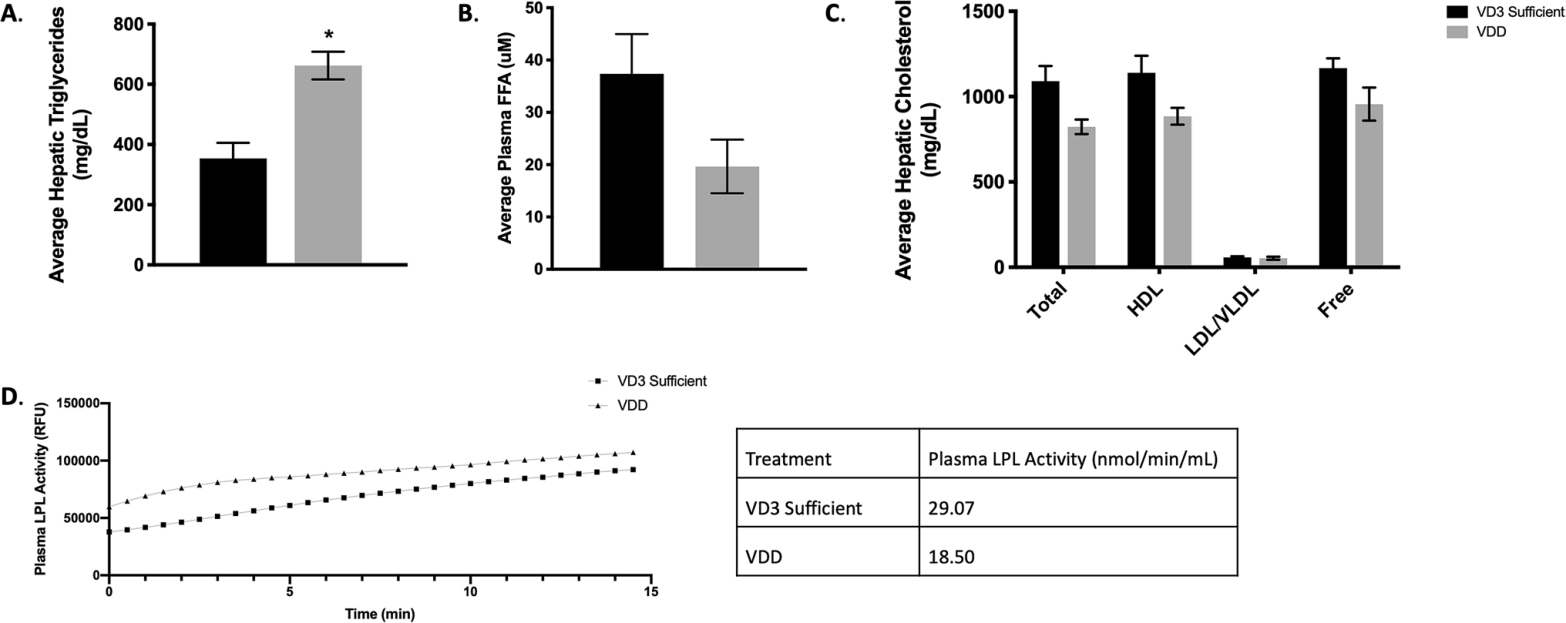
Metabolic homeostasis in VDD liver. (**A**) VDD fish had significantly elevated levels of hepatic triglycerides (662.37 ± 46.25 mg/dL) 6 mpf. Data are represented as mean ± SEM. (**B**–**D**) VDD fish had lower plasma FFA levels (19.67 ± 5.13uM), lower total, HDL, LDL/VLDL, and free hepatic cholesterol (822.7 ± 42.49 mg/dL, 884.9 ± 49.50 mg/dL, 52.7 ± 8.74 mg/dL, 956.2 ± 96.39 mg/dL, respectively) and lower plasma LPL activity (18.5 nmol/min/mL) 6 mpf. Data are represented as mean ± SEM.

Next, hepatic triglycerides, plasma FFA, hepatic cholesterol, and plasma LPL activity were measured to determine the state of metabolic homeostasis in VDD zebrafish liver (Fig. 3). It was observed that VDD zebrafish had significantly elevated levels of hepatic triglycerides (662.37 ± 46.25 mg/dL VDD versus 353.54 ± 51.80 mg/dL VD3 Sufficient) at 6 mpf (Fig. 3A), but lower plasma FFA levels (19.67 ± 5.13uM VDD versus 37.38 ± 7.60 uM VD3 Sufficient) (Fig. 3B), lower total hepatic cholesterol, HDL, LDL/VLDL, and free cholesterol (822.7 ± 42.49 mg/dL, 884.9 ± 49.50 mg/dL, 52.7 ± 8.74 mg/dL, 956.2 ± 96.39 mg/dL, respectively for VDD versus 1090.65 ± 88.80 mg/dL, 1139.90 ± 99.41 mg/dL, 58.60 ± 5.47 mg/dL, 1166.90 ± 57.63 mg/dL, respectively for VD3 Sufficient) (Fig. 3C) and lower plasma LPL activity (18.50 nmol/min/mL VDD versus 29.07 nmol/min/mL VD3 Sufficient) (Fig. 3D).

VDD zebrafish were subsequently assessed for indications of hepatic steatosis. Whole fish sections with H&E indicated normal liver structure in VDD animals (Supplementary Fig. 3). Hepatocyte arrangements in longitudinal array for VDD as well as VD3 sufficient zebrafish, revealed cord-like structures typically two cells thick separated by sinusoidal lumens lined with endothelial cells. There was no significant vacuolation in hepatocyte cytoplasm, and no indication of expanded biliary passageways in control sections. Glycogen storage was assessed through PAS staining and again revealed no significant difference between VDD and VD3 sufficient livers at 6 mpf (Supplementary Fig. 3).

**Figure 4:**
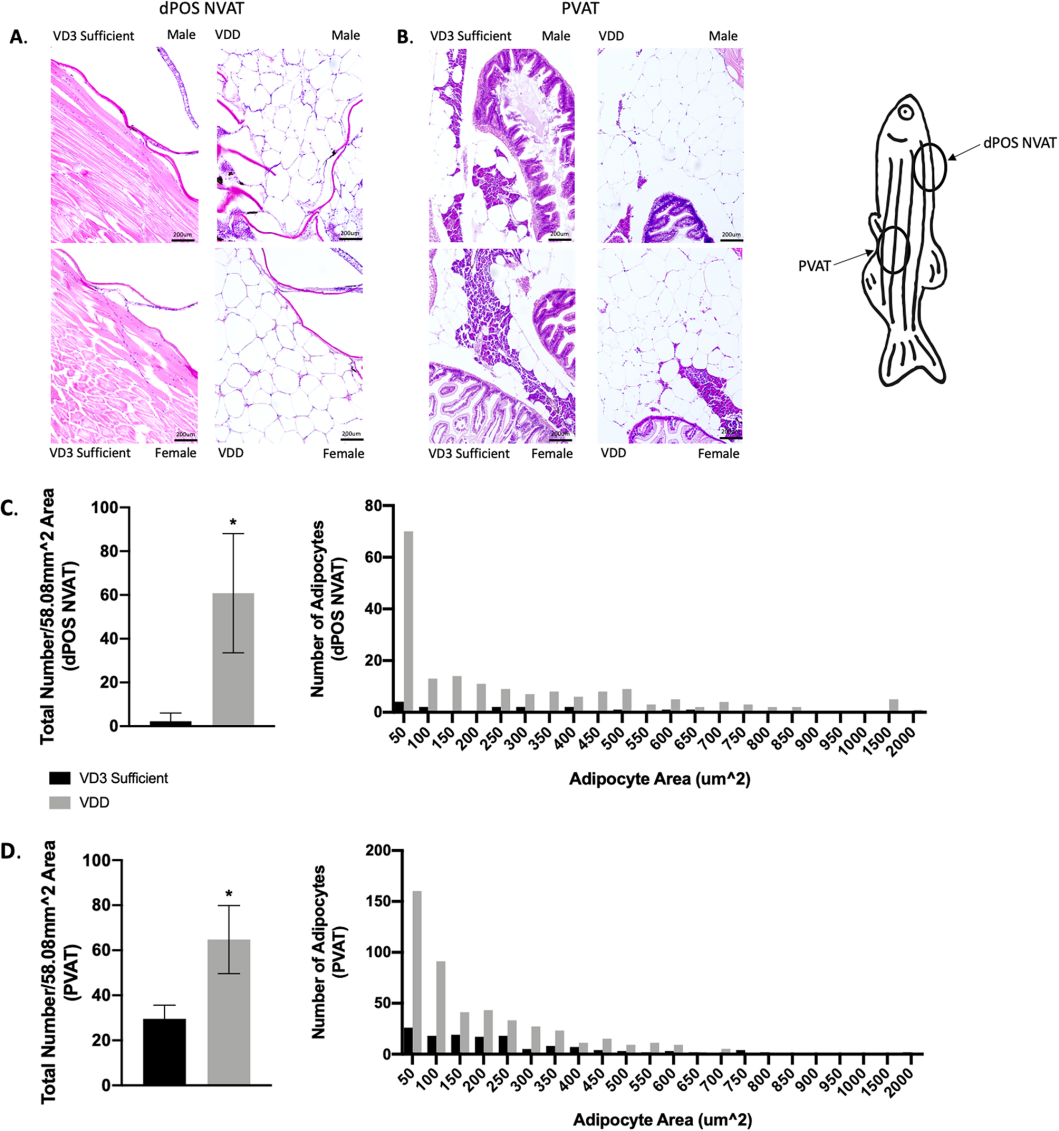
VDD fish demonstrated both hypertrophy and hyperplasia of dorsal paraosseal (dPOS) non-visceral AT (NVAT) and pancreatic visceral AT (PVAT)^31^. (**A**) Representative images of dPOS NVAT (58.08 mm^2^ area). (**B**) Representative images of PVAT (58.08 mm^2^ area). (**C**) VDD fish had hypertrophic and hyperplastic (60.78 ± 9.09 adipocytes) dPOS NVAT compared to VD3 sufficient 6 mpf. Data are represented as mean ± SEM (n = 9). (**D**) VDD fish had hypertrophic and hyperplastic (64.75 ± 15.11 adipocytes) PVAT compared to VD3 sufficient 6 mpf. Data are represented as mean ± SEM (n = 9).

**Figure 5:**
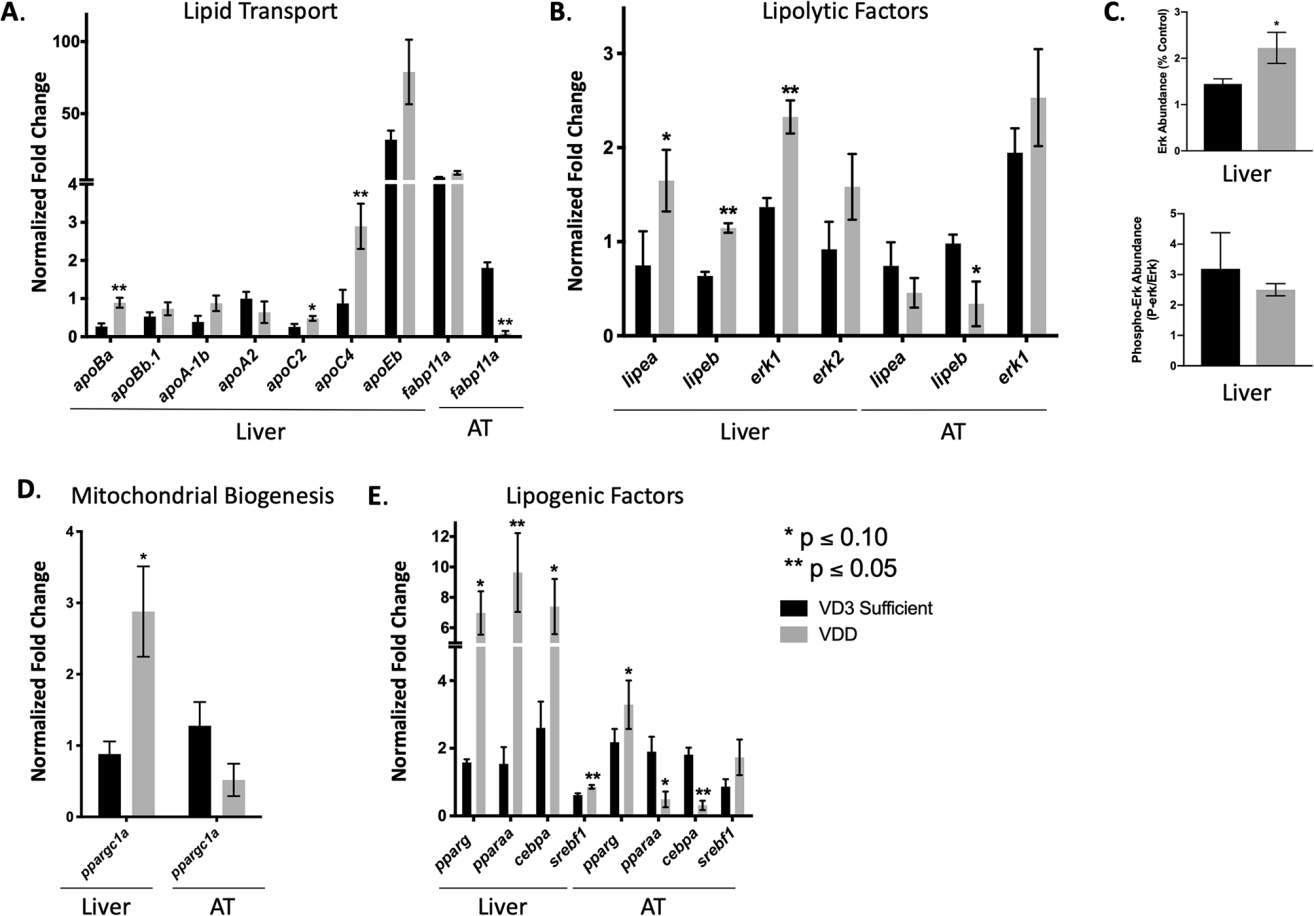
Metabolic dyshomeostasis in VDD AT. (**A**) Elevated expression of lipid transporters in VDD liver 6 mpf. Decreased expression of *fabp11a* in VDD AT 6 mpf. (**B**) Elevated expression of lypolitic factors in VDD liver 6 mpf, with a decrease in *lipea* and *lipeb* in VDD AT. (**C**) Greater abundance of erk1/2 protein in VDD liver 6 mpf, with a decreased in p-erk1/2. (**D**) Suppression of mitochondrial biogenesis in VDD AT compared to VDD liver 6 mpf. (**E**) Elevated expression of lipogenic factors in VDD liver 6 mpf. Elevated expression of *pparg* and *srebf1* in VDD AT 6 mpf with a decrease in *pparaa* and *cebpa*. *See also Figure S3.

Assessment of adipose reserves in whole zebrafish sections revealed that VDD zebrafish exhibited hyperplasia of dorsal paraosseal (dPOS) non-visceral AT (NVAT) (60.78 ± 9.09 adipocytes VDD versus 2.27 ± 1.14 adipocytes VD3 Sufficient) and pancreatic visceral AT (PVAT) (64.75 ± 15.11 adipocytes VDD versus 29.56 ± 6.03 adipocytes VD3 Sufficient), along with hypertrophy of dPOS NVAT and PVAT with an overall trend towards larger adipocytes in the VDD zebrafish (Fig. 4) at 6 mpf^31^. To understand why VDD zebrafish displayed hypertrophy and hyperplasia of AT, but no signs of hepatic steatosis, we evaluated expression of select gene sets for lipid transport, lipolysis, mitochondrial biogenesis, and lipogenesis in both the hepatic and AT tissues (Fig. 5). Apolipoproteins were upregulated in VDD liver particularly *apoBa*, *apoc2*, and *apoc4*; however, while *fabp11a* was not significantly different in the liver, *fabp11a* was significantly attenuated in VDD fish at the AT level (Fig. 5A). Lipolytic factors (*lipea*, *lipeb*, *erk1*, *erk2*) were upregulated in VDD liver and decreased in VDD AT, apart from *erk1* (Fig. 5B). Upregulation of *erk* in VDD zebrafish was confirmed at the protein level; however, liver P-erk1/2 suggests an attenuated decrease in Erk activity in VDD zebrafish (Fig. 5C). *Ppargc1a* was used as a marker of mitochondrial biogenesis, with mRNA levels suggesting depressed mitochondrial biogenesis in VDD AT (Fig. 5D). Upregulation of lipogenic factors (*pparg*, *pparaa*, *cebpa*, *srebf1*) was observed in VDD liver; however, attenuation of *pparaa* and *cebpa* was observed in VDD AT (Fig. 5E).

**Figure 6:**
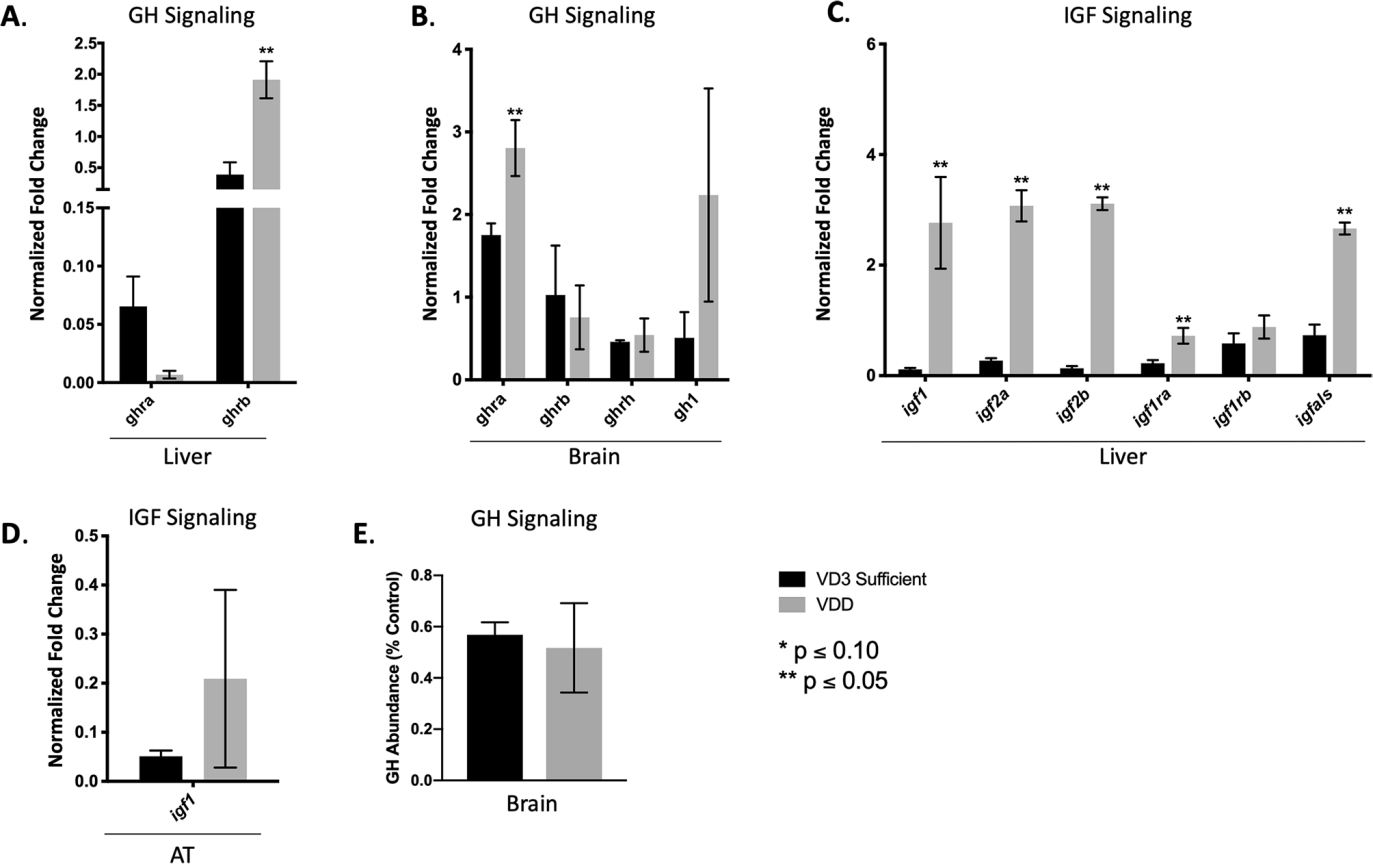
Evaluating GH signaling in VDD liver, AT, and brain. (**A**,**B**) VDD fish had significantly elevated expression of *ghr* in both liver and brain 6 mpf, but no difference in *gh1*. (**C**,**D**) VDD fish had significantly elevated expression of *igf* in liver 6 mpf, with only a slight increase in *igf1* in the AT. (**E**) No difference seen in GH protein abundance in the brain.

**Figure 7:**
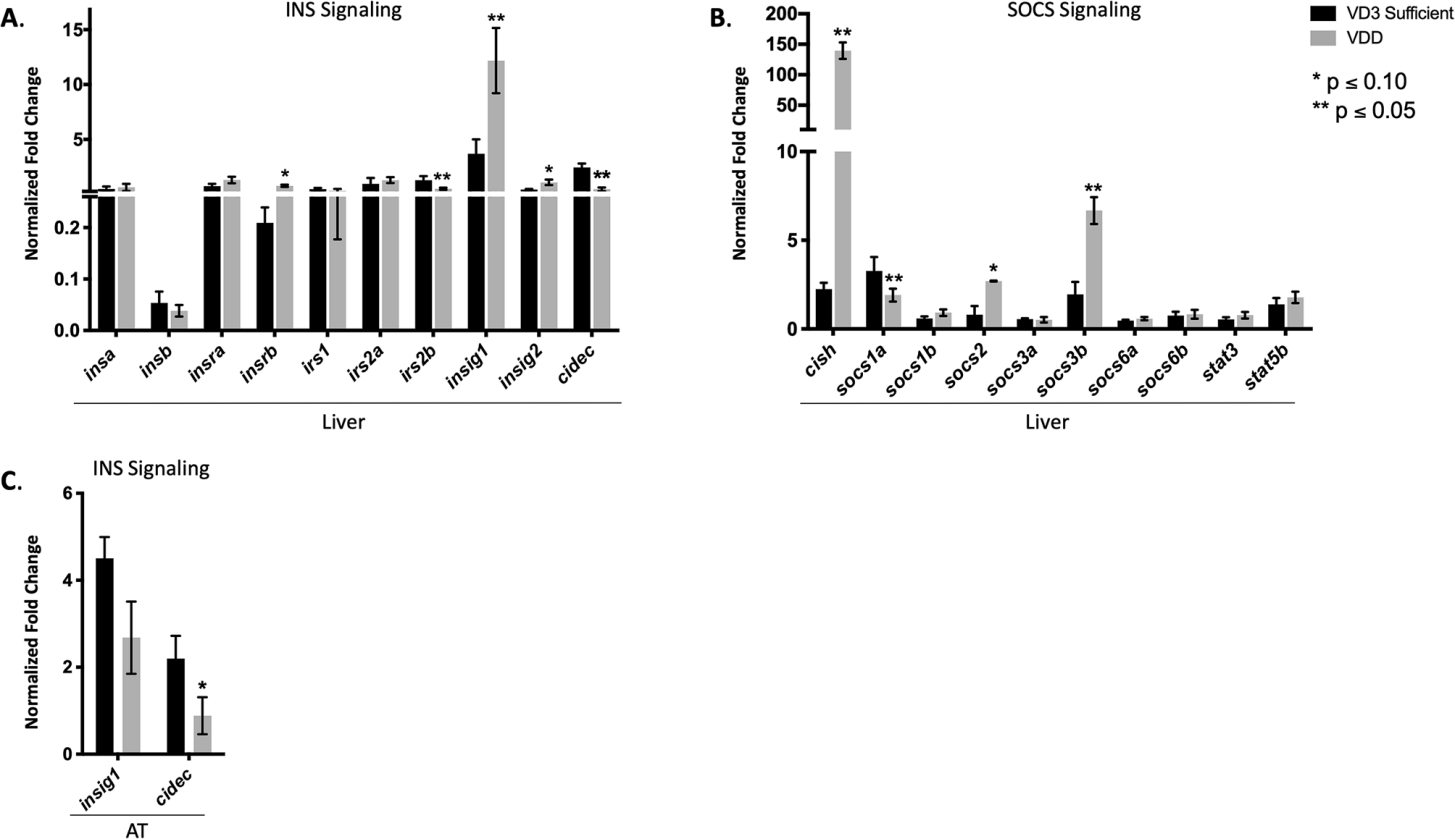
Evaluating INS signaling in VDD liver and AT. (**A**,**B**) VDD fish had significantly elevated expression of *insig1*, *insig2*, with a significant decrease in *cidec* in liver 6 mpf, indicating sustained INS signaling contrary to also having significantly elevated expression of *cish*, *socs2*, and *socs3b*, suppressors of cytokine signaling thought to shut down the INS signaling cascade. (**C**) Downregulation of *insig1* and *cidec* in VDD AT 6 mpf.

**Figure 8:**
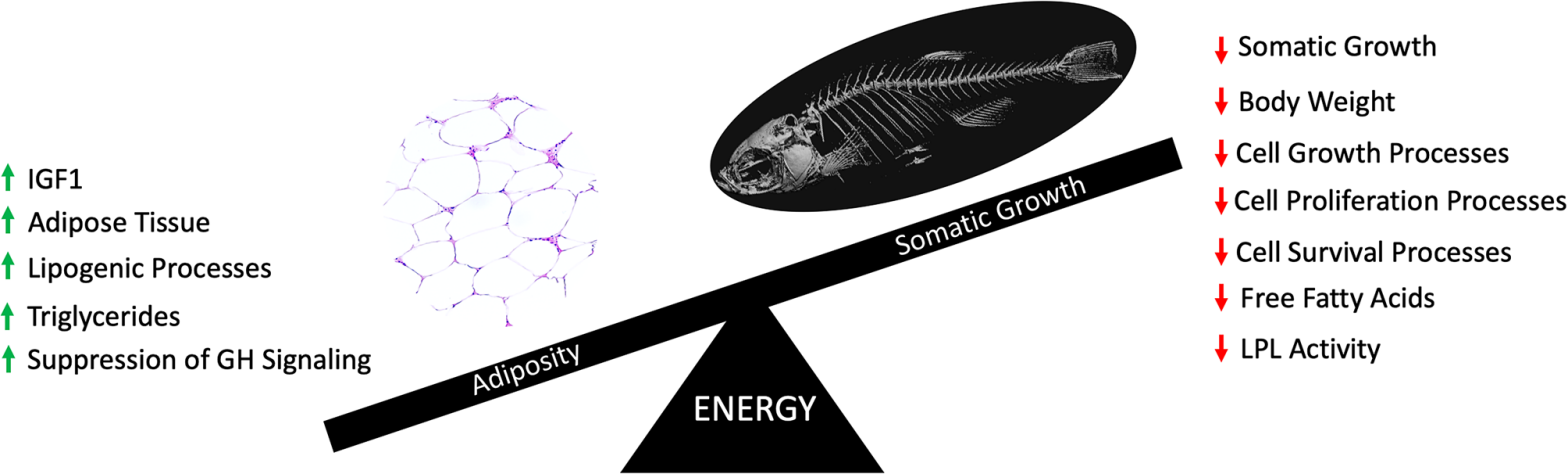
Summarizing the impact of VDD. VDD during early life stage development leads to metabolic dyshomeostasis, where there is an imbalance between somatic growth and adiposity.

Given the observation of stunted growth, GH signaling was investigated in the liver, brain, and AT (Fig. 6). VDD liver demonstrated a downregulation of *ghra* and a significant upregulation of *ghrb* (Fig. 6A). Conversely, VDD brain demonstrated a significant upregulation of *ghra* and a downregulation of *ghrb*, with no significant difference in *ghrh* or *gh1* mRNA expression*,* or Gh protein abundance (Fig. 6B,E). Next, *igf* expression was investigated as a transcriptional target of GH signaling. Contrary to expectations, VDD zebrafish exhibited significantly elevated expression of *igf1*, *igf2a*, *igf2b*, *igf1ra*, and *igfals* in liver at 6 mpf, with only a slight increase in *igf1* in the AT (Fig. 6C,D). The incongruence of stunted growth and elevated *igf* in VDD livers prompted investigation of additional components of GH and INS signaling in VDD and VD3 sufficient livers. Using a combination of RNA-Seq and qPCR we observed significantly elevated expression of *insig1* and *insig2* in liver and a downregulation of *insig1* in AT at 6 mpf (Fig. 7A,C) indicating sustained hepatic INS signaling. Furthermore, VDD zebrafish had a significant decrease in *cidec* in both liver and AT (Fig. 7A,C). Surprisingly, in VDD zebrafish there was a significant elevation in *cish*, *socs2*, and *socs3b*, suppressors of cytokine signaling known to modulate the GH, IGF, and INS signaling cascades (Fig. 7B). Assessments of global transcriptional response with RNA-Seq additionally indicated modulation in *erk* signaling as a putative target linking growth and adipose phenotypes through GH and INS signaling. We found that the *erk* signaling cascade was predicted to be inhibited downstream GH and IGF signaling cascades, indicating that despite elevated levels of growth promoting genes such as *igf1*, downstream cascades responsible for cell growth, cell proliferation, and cell survival were putatively inactive (Supplementary Fig. 4). Supporting this hypothesis, pathway analysis using KEGG predicted *socs* inhibition of insulin receptor activation (Supplementary Fig. 5) and gene set enrichment analysis (GSEA) predicted enrichment (normalized enrichment value of − 3.93) of a network describing metabolic syndrome (Supplementary Fig. 6). Taken together, we suggest a direct link between VDD and disruption of GH and INS signaling, where insufficient levels of vitamin D skew the homeostatic regulation of somatic growth and adipose deposition (Fig. 8).

## Discussion

Vitamin D (1α, 25-dihydroxyvitamin D3) is a steroid hormone traditionally associated with bone homeostasis; however, accumulating evidence suggests a wider biological role for the VD3 signaling axis in metabolic control. Low levels of VD3 during early development have been linked to obesity and metabolic disorders falling under the umbrella of dyslipidemia and adiposity^6–12,29^. To address the role of vitamin D in metabolic control we developed a dietary VDD model with greater capability of mimicking systemic human VDD than previously published genetic knockout models. We demonstrate that dietary induced VDD during early development stimulates a state of metabolic dyshomeostasis that results in stunted somatic growth and central adiposity. Given that VDD impacts roughly one billion individuals globally, and obesity has tripled since 1975 impacting nearly two billion individuals globally, it is critical to investigate putative linkages between these populations^1,4^.

Unlike mammals, most teleost fish including zebrafish, are unable to synthesize vitamin D from the sun through the conversion of provitamin D (7-dehydrocholesterol) in the skin. Rather they are dependent upon dietary sources of vitamin D3 (cholecalciferol) and vitamin D2 (ergocalciferol)^32^. Here we took advantage of the dietary dependency of this nutrient and demonstrate that zebrafish fed a vitamin D null diet exhibited significantly lower (~ 5000 fold) whole-body levels of 1,25(OH)2D3 compared to fish feed a vtiamin D sufficient diet. Together our LC/MS/MS data commbined with skeletal morphology and expression of vitamin D metabolic genes, demonstrate the efficacy of our dietary model to induce VDD.

Interestingly early studies examining metabolic activation of vitamin D to it’s active form 1,25(OH)2D3, demonstrated a marked attenuation in circulating plasma levels 25(OH)D3 in fish compared to other species^32^. Our LC/MS/MS results were consistant with this observation with low or undectitable levels of 25(OH)D3 observed in our VD3 sufficient or VDD fish respectivley. It is hypothesized that low systemic levels of 25(OH)D3 are likely due to the fact that fish hepatic tissues can facilitate both the 1α- and 25-hydroxlyation reactions forming 1,25(OH)2D3 without the need to transport to the kidney^32,33^. This function may inturn provide an opportunity for more efficient conversion of 25(OH)D3 to 1,25(OH)2D3 resulting in lower overall systemic levels of 25(OH)D3 compared to 1,25(OH)2D3. Indeed our data demonstrates over a 1000 fold concentration diference between 25(OH)D3 and 1,25(OH)2D3 from whole body extracts of fish fed a VD3 sufficient diet.

With induction of VDD by six months of age, both male and female VDD zebrafish exhibited stunted growth, indicated by shorter SL, lower SGR, and lower body weight and BWI, within 4 months on a vitamin D null diet. Re-introducing vitamin D to the VDD population at 6 mpf promoted growth within 2 weeks; however, VDD + VD3 zebrafish were unable to catch up in SL and weight of the vitamin D sufficient and LD groups. Despite displaying elevated levels of central adiposity, VDD zebrafish did not demonstrate hyperphagic feeding behavior or signs of hepatic steatosis and/or glycogen storage which is common in studies of zebrafish feed high fat diets^34,35^. Conversely, VDD zebrafish exhibited significantly elevated levels of hepatic triglycerides concurrent with alterations in hepatic and plasma lipid indices; findings consistent with an underlying obese phenotype^36^. Interestingly, VDD zebrafish also exhibited significantly increased expression of apolipoproteins in the liver supporting lipid transport of triglycerides out of the liver and the observed absence of hepatic steatosis. Furthermore, VDD zebrafish exhibited decreased LPL activity, concurrent with epidemiological studies demonstrating an association between low vitamin D levels and reduced LPL activity^22^. This data indicated that the observed central adiposity was more likely due to metabolic dyshomeostasis at the AT level rather than at the hepatic level.

Given our combined stunted growth and obese phenotypes, we sought to determine if GH signaling was modulated in our model. Previous studies using adipose tissue-specific GHR knockout (Ad-GHRKO) mice demonstrate similar disruptions in metabolic homeostasis^37^. Ad-GHRKO knockout mice exhibit no disruption in global GH or IGF signaling, or changes in serum glucose levels, and axial growth^37^. Rather, these KO mice demonstrate significant increases in body weight, hypertrophy and hyperplasia of AT, decreased lipolysis, and resistance to hepatic steatosis, phenotypes much in line with our VDD zebrafish^37^. Similarly, *vizzini* zebrafish mutants, which contain a premature stop codon in *gh1*, demonstrate no disruption in global IGF signaling, but exhibit stunted growth, hyperplasia and hypertrophy of AT, and decreased mobilization of AT during periods of starvation^28^. Our model appears to be consistent with such phenotypes representing disruption of AT GHR. We found that our VDD zebrafish had both hypertrophy and hyperplasia of dPOS NVAT and PVAT at 6 mpf consistent with the *vizzini* zebrafish mutants and GH-deficient zebrafish lines where loss of functional *gh1* led to a higher abundance of visceral and subcutaneous AT compared to controls^29^. One possible explanation for the hypertrophic phenotype observed could be the significant attenuation of *fabp11a* in the VDD AT, indicating attenuation of fatty acid efflux. We further found that VDD zebrafish were stimulating lipogenesis at the hepatic level, but mRNA abundance of lipid transporters and lipolytic factors suggested packaging and transport of lipid to AT, which supported the absence of hepatic steatosis. Downregulation of *ppargc1a*, *lipea*, *lipeb*, and *insig1* in VDD AT indicated suppressed mitochondrial biogenesis and lipolytic activity potentially due to disruption in GH/IGF signaling at the AT level. Zebrafish *cyp2r1* knockouts demonstrate a similar pattern in *ppargc1a* expression concurrent with stunted growth, central adiposity, an upregulation in *igf1* in both liver and AT, and no evidence of hepatic steatosis^27^. These models are in direct comparison to zebrafish fed high fat diets which do not display truncated growth but do display significant obesity and hepatic steatosis consistent with dyslipidemia^34,35^.

In the *cyp2r1* mutant zebrafish model, decreased activation in vitamin D biosynthesis is linked to increased *ghra* expression in the liver and AT, along with elevated levels of GH protein in pituitary^27^. Similarly, research using VDD German Landrace piglets found that despite being stunted in growth and having lower body weights, these VDD piglets also had elevated levels of GH^38^. To understand if VDD fish exhibited alterations in GH signaling, we assessed GH markers in the liver, brain, and AT. We found that VDD liver and brain demonstrated opposing expression patterns for *ghra* and *ghrb*. It is known that as a result of receptor sub-functionalization, *ghra* (GHR1) responds to GH and somatolactin as ligands, while *ghrb* (GHR2) responds only to GH activation^39^. Downregulation of *ghra* in the liver and *ghrb* in the brain, could represent a compensatory mechanism unique to our model.

Aside from its own autocrine activity, the IGF signaling cascade is a major target of GH signaling, where GH increases *igf1* levels and *igf1* negatively feeds back to decrease GH levels^40,41^. Together, IGF and GH work to maintain energy homeostasis by balancing somatic growth and adiposity, with elevated levels of IGF correlating with increased somatic growth^39,42^. Contrary to predictions, both VDD and *cyp2r1* mutant zebrafish displayed elevations in *igf1* despite also demonstrating stunted growth^27^. Knowing that compensatory autocrine activity exists for *igf1* at tissue-specific levels, one hypothesis is that despite sustained *igf1* activity in liver and AT, VDD zebrafish may have suppressed *igf1* activity in bone. Ohlsson et al., 2009 demonstrated that bone-specific IGF-1 KO mice were unable to undergo longitudinal growth despite sustained *IGF1* levels in liver and other tissues^40^. Further research suggests that elevated *igf1* levels can be found in obese states, due to sustained *igf* activity at the AT level^43,44^.

To understand local sources of *igf1* and potential regulatory mechanisms of the GH/IGF signaling axis, *ins* and *socs* signaling were investigated. We found that VDD zebrafish demonstrated sustained expression of genes comprising the INS signaling pathway suggesting no indication of hepatic insulin resistance. Surprisingly, there was also a significant elevation in suppressors of cytokine signaling in the liver, known to modulate/inhibit GH, IGF, and INS signaling cascades^45,46^. Based on our RNA-Seq dataset, we demonstrate a direct role for Socs in inhibition of INS signaling, and identified Erk signaling as a putative target of Socs inhibition downstream of both GH and IGF signaling cascades. Further assessment of *erk* mRNA levels indicated that VDD zebrafish exhibited significant upregulation of *erk1/2* in the liver and upregulation of *erk1* in the AT. Upregulation of *erk* in VDD zebrafish was confirmed at the protein level however, an attenuated liver P-erk1/2 signal suggests an overall putative decrease in Erk activity. These observations suggest that overexpression of *socs* and modulation of *erk* may contribute to suppression of cell growth, cell proliferation, and cell survival in the VDD zebrafish which may link VDD, stunted growth, and adiposity. Furthermore, GSEA analysis suggested enrichment of genes associated with metabolic syndrome in our VDD fish. Metabolic syndrome is widely characterized by obesity, insulin resistance, hypertension, and hyperlipidemia^47^. Ultimately, metabolic syndrome is considered a state of metabolic dyshomeostasis, where energy utilization becomes off-balance, similar to what was observed in our VDD fish^47^.

In our model, we demonstrate that following VDD, metabolic dyshomeostasis occurs in the form of stunted growth and adiposity. We found elevated levels of *igf1*, hypertrophy and hyperplasia of AT, enhanced lipogenic processes, elevated hepatic triglycerides, and suppression of GH signaling, all of which suggest AT storage. Concurrently, we found decreased somatic growth, decreased body weight, suppressed cell growth, cell proliferation, and cell survival signaling, lower levels of FFAs, and reduced LPL activity, all of which further suggest AT storage, rather than catabolism for growth promoting processes. Based on our data and published models of GH disruption, it appears that VDD may disrupt the coordination of GH and AT signaling through inactivation by *socs*. Vitamin D has been shown to regulate *gh1* transcription via vitamin D response elements (VDREs) found in the *gh1* promoter region^48,49^. Vitamin D has also been shown to inhibit *socs* signaling as another mechanism of GH regulation^50^. This relationship could demonstrate a direct link between VDD and disruption of GH signaling, where insufficient levels of vitamin D skew the homeostatic regulation of somatic growth and adipose deposition.

## Methods

### Zebrafish housing and care

All zebrafish (Danio rerio) in this study were housed and cared for according to standard protocols approved by the North Carolina State University (NC State) Institutional Animal Care and Use Committee. Adult zebrafish were maintained at appropriate densities in 9L tanks as part of a recirculating aquatics system under a 14:10 hr light:dark cycle. Water temperature was maintained at 28.5 ± 0.5 °C with a pH between 6.8 and 7.5. To ensure that VDD zebrafish did not exchange water with LD controls or VD3 sufficient fish, they were kept in their own quarantine system set to the same parameters as the main system.

### Cohort generation

To generate F0 cohorts, ABxAB zebrafish were co-housed and raised on a standard larval laboratory diet (11.5 iu/g VD) until 2 months of age. At the two-month time point, a random sample of 61–83 juvenile zebrafish were assessed for standard length (SL) to determine the average SL for the population. Based on the average SL, any fish that was ± 2 standard deviations (SD) away from the mean was removed from the population. The remaining fish were equally divided and placed into nine, 9L, mixed-gender tanks. The tanks were grouped in sets of three so that there were three tanks per diet. Upon transfer into their new housing, the 2-month old zebrafish began their new diet: VD3 null diet (0 iu/g VD3), VD3 enriched diet (400,000 iu/g VD3), or standard LD control (1.4 iu/g VD3). The zebrafish were kept on their designated engineered diet throughout the rest of their lifespan. Only male zebrafish were used in molecular studies to account for the impact of sex differences on vitamin D deficiency, growth, and adiposity. LD populations containing 1.4 iu/g VD3 were used for data normalization in specified studies since growth and development tracked well with the VD3 sufficient group.

### Growth rate

Growth rate was taken biweekly starting at 2 mpf and ending at 6 mpf (2 mpf, 2.5 mpf, 3 mpf, etc.). Twenty fish/diet (mixed gender) were randomly selected and weighed using a Denver Instrument M220-D balance. SL measurements were then taken using a standard centimeter ruler. SL was determined to be the distance from the snout to the caudal peduncle as described by Parichy et al.^51^. SL (cm) and weight (g) were used to calculate body weight increase (BWI) and specific growth rate (SGR) according to the Mehrad et al., protocol^52^.

### Quantification of 1,25(OH)2D3 and 25(OH)D3

Vitamin D metabolites were analyzed on n = 5 male fish per diet as previously described^53^. Briefly, a solution of 400 µL methanol/acetonitrile/formic acid (76/19/5) and 10 µL internal standard solution (20 nM 1α,25(OH)_2_VitD_3_-*d6* and 200 nM 25(OH)VitD_3_-*d6*) were added to zebrafish tissue (50–100 mg), followed by homogenization avoiding direct light. The tissue homogenate was stored for 24 hr at -80 °C. On day two, the homogenate was centrifuged for 5 min at 10,000 g, and the supernatant was transferred into 2 mL centrifuge tube. The remaining tissue was washed by additional 200 µL methanol/acetonitrile/formic acid (76/19/5). After centrifugation, the supernatants (~ 600 µL) were combined, followed by the addition of 600 µL 0.4 M K_2_HPO_4_. Further LLE-SPE extraction and PyrNO derivatization, as well as LC/MS/MS analysis were reported previously^53^. Analyst software 1.6.3 was used to quantify each vitamin D metabolites.

### Histology/histopathology & adipocyte morphology

Zebrafish were euthanized in ice cold water according to the protocol outlined by Wilson and colleagues^54^. The ventral abdomen of each fish was cut open to allow optimal fixation of organs. Fish were fixed whole in 10% neutral buffered formalin (NBF) for at least 24 h, embedded in paraffin, sectioned at 5um thickness, and stained with hematoxylin and eosin (H&E) or Periodic Acid-Schiff (PAS) at the NC State College of Veterinary Medicine (Raleigh, NC). Sagittal sections and step sections were analyzed for histopathology and morphometric studies. H&E staining was performed on four separate populations with an n = 3–10 for each diet depending on total population size. PAS staining was completed on n = 5 fish per diet across one population. For morphometry, H&E sagittal and step sections of pancreatic and subcutaneous fat were photographed at 10X objective using an Olympus BX microscope. The images of nine fish per diet were grouped by sex and specified fat depots were analyzed in Fiji (ImagJ version: 2.0.0-rc-64/1.51 s) following an adjusted version of the Parlee et al. protocol^55^.

### Triglycerides

Hepatic triglycerides were measured using a Triglyceride Quantification Assay Kit (Abcam, Cambridge, MA). Four samples containing two pooled livers each were analyzed per dietary group. Reagents and standards were prepared prior to use according to the manufacturer’s protocol. Tubes of liver were weighed to ensure there was at least 100 mg of tissue per tube. Thawed livers were washed in cold 1 × phosphate buffered saline (PBS) (Caisson Labs, Smithfield, UT) and 500 uL of 5% NP-40/ddH_2_0 (Sigma-Aldrich, Milwaukee, WI) was added to each tube. The samples were homogenized using a bio-vortexer (BioSpec Products, Bartlesville, OK). Once fully homogenized, 500 uL of 5% NP-40/ddH_2_0 was added to bring the volume up to 1 mL. The samples were then heated for two cycles in a 100 °C water bath for 5 min and centrifuged for 2 min at 15,000 rpm. Lastly, the samples were diluted in 9 mL of ddH_2_0. All reagents, standards, and samples were brought to room temperature prior to plating in a black polystyrene, 96-well, flat bottom plate (Corning, Corning, NY). Once the reactions were prepared and added to the appropriate wells, the plate was mixed and placed in a 28.2 °C incubator for 60 min, protected from light. Triglyceride levels were measured using a FLUOstar Omega Microplate Reader (BMG LabTech, Cary, NC) and associated software. Output was measured at Ex/Em = 544/590 nm. Results were calculated based on the standard curve equation and the provided triglyceride concentration equation.

### LPL activity

Plasma LPL activity was measured using a Lipase Activity Assay Kit (Abcam, Cambridge, MA). Each 1.5 mL microcentrifuge tube contained pooled plasma from 4–8 males. Reagents, positive controls, and standards were prepared prior to use according to manufacturers’ protocol. The plasma was diluted 1:10 with 1 × Assay Buffer prior to starting assay. Once the samples were prepared, all reagents, standards, and samples, except for the Lipase Positive Control, were brought to room temperature prior to plating in a black polystyrene, 96-well, flat bottom plate (Corning, Corning, NY). Once the reactions were prepared and added to the appropriate wells, the plate was placed in a FLUOstar Omega Microplate Reader (BMG LabTech, Cary, NC) set to 37 °C and fluorescence was measured every 30 s for 15 min. Output was measured at Ex/Em = 355/520. Results were calculated based on the standard curve equation and provided lipase activity equation.

### Cholesterol

Hepatic cholesterol (total, free, HDL, LDL/VLDL) was measured using a HDL and LDL/VLDL Cholesterol Assay Kit (Abcam, Cambridge, MA). Each 1.5 mL microcentrifuge tube contained two pooled livers and there were four tubes per diet group. Reagents and standards were prepared prior to use according to manufacturers’ protocol. Tubes of liver were weighed to insure there was at least 10 mg of tissue per tube. Thawed livers were washed in cold 1 × PBS (Caisson Labs, Smithfield, UT) and 100 uL of cholesterol assay buffer was added to each tube. The samples were homogenized using a bio-vortexer (BioSpec Products, Bartlesville, OK) and kept on ice. The homogenized livers were then centrifuged at 13,000 rcf for 10 min at 4 °C. The supernatants were removed and kept on ice to be used as the total cholesterol samples. For each of the diets, 50 uL of the total cholesterol fraction was removed and added to 50 uL of 2 × precipitation buffer to eventually become the HDL fraction and the LDL/VLDL fraction according to manufacturer’s protocol. Once the samples were prepared, all reagents, standards, and samples were brought to room temperature prior to plating in a black polystyrene, 96-well, flat bottom plate (Corning, Corning, NY). Once the reactions were prepared and added to the appropriate wells, the plate was mixed and placed in a 28.2 °C incubator for 60 min, protected from light. Cholesterol levels were measured using a FLUOstar Omega Microplate Reader (BMG LabTech, Cary, NC) and associated software. Output was measured at Ex/Em = 544/590 nm. Results were calculated based on the standard curve equation and the provided cholesterol concentration equation.

### Free fatty acids

Plasma FFA concentrations were measured using a Free Fatty Acid Fluorometric Assay Kit (Abcam, Cambridge, MA). Each 1.5 mL microcentrifuge tube contained pooled plasma from 10–15 males. Reagents, positive controls, and standards were prepared prior to use according to manufacturers’ protocol. Once the samples were prepared, all reagents, standards, and samples were either brought to room temperature or, if specified, kept on ice prior to plating in a black polystyrene, 96-well, flat bottom plate (Corning, Corning, NY). Prior to adding the Developer to the wells, the plate was covered and incubated at 37 °C for 30 min. Once the Developer was added, the plate was covered and incubated at 37 °C for 15 min. The plate was then uncovered and placed in a FLUOstar Omega Microplate Reader (BMG LabTech, Cary, NC) set to 37 °C and fluorescence was measured. Output was measured at Ex/Em = 544/590. Results were calculated based on the standard curve equation and provided free fatty acid concentration equation.

### qPCR

Quantitative Real-Time PCR (qPCR) was used to measure targeted gene expression in male zebrafish liver, brain, and AT at 6 months of age. cDNA was synthesized from total RNA using 10 × random primers, 10 × reverse transcription buffer, MultiScribe Reverse Transcriptase, and 10 mM deoxynucleotide triphosphates from a High Capacity cDNA Reverse Transcription Kit (Applied Biosystems, Foster City, CA) along with RNasin RNase Inhibitor (Promega, Madison, WI) to make a 20 uL reaction. Gene sequences specific to zebrafish were first identified using Ensembl, release 88 and 89. Primer sequences were designed using Primer3web, version 4.1.0. Primers were ordered from Integrated DNA Technologies, Inc. (Coralville, Iowa). See Supplementary Table 1 for a full list of primer sequences. All primers were tested for efficiency across a range of template cDNA dilutions (1/10–1/500) prior to use for experimental purposes. Gene expression patterns in vitamin D deficient, vitamin D sufficient, and control zebrafish liver samples were quantified using an Applied Biosystems 7300 real time PCR machine. Biological replicates (n = 3–4/diet) were plated in triplicates and amplified in a 96-well, clear Olympus PCR plate (Genesee, Morrisville, NC). Each well contained a 20 uL mixture: 6.8uL of UltraPure water (Invitrogen, Marietta, OH), 0.8uL of 10 uM forward primer, 0.8 uL of 10 uM reverse primer, 1.6 uL of cDNA at a 1:10 dilution, and 10 uL of iTaq Universal SYBR Green Supermix (Bio-Rad, Hercules, CA). Once the PCR machine was primed, each reaction occurred under the following conditions: (1) 50 °C for 2 min, (2) 95 °C for 10 min, (3) 95 °C for 15 s followed by 60 °C for 1 min (repeated 40 times). This cycle was followed by a dissociation stage which ensured primer specificity and confirmed the absence of primer dimerization: (4) 95 °C for 15 s, 60 °C for 1 min, 95 °C for 15 s, 60 °C for 1 min. Individual threshold cycle values (C_t_) were determined for each reaction by the ABI 7300 System SDS Software and relative fold change differences for each gene across each sample was calculated according to the ΔΔC_t_ method^56,57^. Gene expression was normalized to *efla* as the housekeeping gene.

### Western blots

Protein content in liver and brain was determined using a western blot assay. Zebrafish liver and brain samples, previously dissected and stored at − 80 °C, were placed on ice to thaw. Each 1.5 mL microcentrifuge tube contained two pooled livers or brains, and there were four tubes per diet group. Tissues were washed 3 × with 500 uL of cold PBS. After the third wash, 500 uL of RIPA buffer, 5 uL of phosphatase inhibitors, and 5 uL of protease inhibitors were added to each tube immediately followed by three 15 s homogenization intervals using an Polytron 2100 homogenizer (Kinematica, Luzern, Switzerland). Samples were then centrifuged at 12,000 rpm for 20 min at 4 °C. Supernatant was stored at − 20 °C. Protein concentrations were determined using a Pierce BCA Protein Assay Kit (Thermo Fisher, Raleigh, NC) following manufacturers protocol. 15-20ug of protein for each sample was loaded onto a Novex 12% Tris–Glycine Mini Gel (Thermo Fisher, Raleigh, NC) and separated by electrophoresis. After separation, the proteins were transferred to a PVDF membrane, blocked in 5% BSA/TBS-TT, and incubated at 4 °C overnight with primary antibody in 5% BSA. Primary antibody concentrations varied according to manufacturer’s protocol and our optimization: Chicken Growth Hormone Antibody (1:200, BioVision, Milpitas, CA); p44/42 MAPK (Erk1/2) Antibody (1:1000, Cell Signaling Technology, Danvers, MA); Phospho-p44/42 MAPK (Erk1/2) (Thr202/Tyr204) Antibody (1:1000, Cell Signaling Technology, Danvers, MA); B-Actin (AC-15) (1:1000, Santa Cruz Biotechnology, Inc., Santa Cruz, CA). After incubation with primary antibody, the membrane was washed with TBS-TT and incubated with secondary antibody (1:8000) in 5% BSA for 1 hr at room temperature. Proteins were detected using the GE Healthcare ECL Detection Kit (Thermo Fisher, Raleigh, NC) following manufacturer’s protocol. Images were obtained using the Amersham Imager 680 (GE Healthcare, Marlborough, MA) and densitometry was performed using ImageQuant TL 1D v8.2.0 (GE Healthcare, Marlborough, MA). Total protein was normalized to b-actin and phospho-protein was normalized to total protein.

### Statistical analysis

All data are presented as mean ± SEM unless otherwise indicated as SD. Significance was determined by statistical t-tests using GraphPad Prism software (v8) and Excel. Statistical significance was indicated by *p* ≤ 0.10 (*) and *p* ≤ 0.05 (**). All gene expression data was normalized to LD which was set to a fold-change of 1.

## Limitations of the study

While zebrafish serve as an excellent model of metabolic disease there are limitations to its utility. Specifically, limitations were encountered experimentally due to the size of the organism and the availability of zebrafish-specific reagents including antibodies. A major limitation was the terminal nature of blood collection and the challenges with collecting sufficient volumes for whole blood and plasma analyses. Large numbers of fish were sacrificed and pooled for both the FFA and LPL activity assays which impacted the ability to achieve statistical significance. Similarly, blood glucose tests using a glucometer were inconclusive. Without an available ELISA to measure insulin in zebrafish, the authors were unable to measure plasma insulin levels which prevented a complete understanding of insulin signaling differences across treatment groups. The overall size of zebrafish brain forced the authors to measure GH protein and *gh1* gene expression using whole brain rather than pituitary, which would have been the sample of choice. Lastly, due to the loss of transparency by 6 mpf, live-staining of AT to analyze whole body lipid deposition and localization was not possible for this study. Overall, the authors were able to begin defining the underlying mechanisms linking VDD to stunted growth and central adiposity despite these limitations, but future work is necessary to understand the initiating events prior to the onset of the phenotypes.

## Supplementary information

Supplementary Information.

Supplementary Figure 1.

Supplementary Figure 2.

Supplementary Figure 3.

Supplementary Figure 4.

Supplementary Figure 5.

Supplementary Figure 6.
