# Supplementary material for: Vitamin D deficiency serves as a precursor to stunted growth and central adiposity in zebrafish
Source: Sci Rep. 2020 Sep 29;10:16032. doi: 10.1038/s41598-020-72622-2 (PMC7524799; doi:10.1038/s41598-020-72622-2)
Supplement: Supplementary file 1 — Supplementary Information. [file 41598_2020_72622_MOESM1_ESM.docx]

**Vitamin D deficiency serves as a precursor to stunted growth and central adiposity in zebrafish**

Megan M. Knuth^1*^, Debabrata Mahapatra^2^, Dereje Jima^3,4^, Debin Wan^5^, Bruce D. Hammock^5^, Mac Law^6^, Seth W. Kullman^1,4*^

^1^Toxicology Program, Department of Biological Sciences, North Carolina State University, Raleigh, NC 27606, USA.

^2^Comparative Biomedical Sciences, College of Veterinary Medicine, North Carolina State University, Raleigh, NC 27606, USA.

^3^Bioinformatics Research Center, North Carolina State University, Raleigh, NC 27606, USA.

^4^Center for Human Health and the Environment, North Carolina State University, Raleigh, NC 27606, USA.

^5^Department of Entomology and Nematology and University of California Davis Comprehensive Cancer Center, University of California Davis, Davis, CA 95616, USA.

^6^Department of Population Health and Pathobiology, College of Veterinary Medicine, North Carolina State University, Raleigh, NC 27606, USA.

*Corresponding authors: Seth Kullman, swkullma@ncsu.edu; Megan Knuth, [mmknuth@ncsu.edu](mailto:mmknuth@ncsu.edu); mailing address - Campus Box 7633, Raleigh, NC 27695-7633, telephone 919.515.4378

**Supplemental Data**

**Methods**

*MicroCT*

MicroCT scans were performed by Dr. Hong Yuan and Jonathan Frank at the University of North Carolina (Chapel Hill, North Carolina) (BRIC Small Animal Imaging Facility). High resolution CT was performed on VDD and sufficient zebrafish (n=4/diet) using a microCT imaging system (mCT-40, SCANCO Medical, Bruttisellen, Switzerland). The system was operated at 45 kVp, 88 uA current, 200 msec of integration time, and 500 projections. CT images were reconstructed using the Feldkamp algorithm with 12 micrometer of voxel size. Scan field of view was 12 mm in axial length to cover the regions of interest. Regions of interest were drawn around each sample and relevant bone and cartilage voxels were segmented via SCANCO software. Total volume (entire sample), segmented volume (bone and cartilage), and surface area in addition to the average density (HU) for total volume and segmented voxels were calculated and reported.

*Quantification of Calcium*

Whole fish calcium levels were detected using a Thermo Scientific iCAP RQ ICP-MS instrument. Five male zebrafish replicates from each dietary group were submitted for sample processing at 6 mpf. To a 30mL teflon reaction vessel, 1 fish + 8mL of concentrated HNO_3_ and 2mL of 30% H_2_O_2_ were added and samples were digested overnight. The next morning 1 mL of digestate and 250 μL of a 10ppm Ge IS were added and DI water was filled to line of a 50mL volumetric flask for the 1:50 dilution required for VDD samples. Alternatively, 20 μL of digestate and 250 μL of a 10ppm Ge IS were added and DI water was filled to line of a 50mL volumetric flask for the 1:2500 dilution required for LD and VD3 sufficient samples. All iCAP-RQ ICP-MS measurements and quantitation were made in the Molecular Education, Technology, and Research Innovation Center (METRIC) at North Carolina State University (Raleigh, NC).

*Feeding Rate*

Feeding rate was taken every month starting at 2 mpf and ending at 6 mpf (2 mpf, 3 mpf, 4 mpf, etc.). To measure food intake, 16 fish from each diet (mixed gender) were placed into a new 9L tank at 2 mpf and were given one week to acclimate to their new diet prior to starting the feeding rate experiment. To measure food intake, we followed the Yang et. al protocol.^1^ Prior to starting the experiment, the fish underwent a 24-hour starvation. On the morning of the start of the experiment, all 16 fish from each tank were weighed (g) using a Denver Instrument M220-D balance. For 4-5 days, three times a day, the fish were fed to satiation. Each feeding was around 3-4 hours apart. Food intake was measured using the following equation: Feeding rate (%/d) = 100 * (food intake/initial body weight/days) as described by Yang et. al., 2017.^1^

*Catch-Up Growth*

Catch-up growth was determined following the growth rate protocol previously described, with measurements taken biweekly from 2-11 mpf. At the 6 mpf time-point a subset of VDD fish were given the vitamin D supplemented diet to determine if vitamin D supplementation could promote growth and fat loss after the stunted and obese phenotypes were present.

*RNA Seq*

Illumina RNA library construction and RNA sequencing was completed at the NC State Genomic Sciences Laboratory (Raleigh, NC). Prior to library construction, total RNA was extracted from vitamin D deficient, sufficient, and control male zebrafish livers (n=4/diet), and analyzed for purity, concentration, and integrity using an Agilent 2100 Bioanalyzer (Agilent Technologies, Cary, NC) and associated software with a RNA 6000 Nano Chip (Agilent Technologies, Cary, NC). Purification of messenger RNA (mRNA) was performed using the oligo-dT beads provided in the NEBNExt Poly(A) mRNA Magnetic Isolation Module (New England Biolabs, Ipswich, MA). Complementary DNA (cDNA) libraries for Illumina sequencing were constructed using the NEBNext Ultra Directional RNA Library Prep Kit (NEB) and NEBNext Mulitplex Oligos for Illumina (NEB) using the manufacturer-specified protocol. Briefly, mRNA was chemically fragmented and primed with random oligos for first strand cDNA synthesis. Second strand cDNA synthesis was then carried out with dUTPs to preserve strand orientation information. The double-stranded cDNA was then purified, end repaired and “a-tailed” for adaptor ligation. Following ligation, the samples were selected from a final library size (adapters included) of 400-550 bp using sequential AMPure XP bead isolation (Beckman Coulter, USA). Library enrichment was performed and specific indexes for each sample were added during the protocol-specified PCR amplification. The amplified library fragments were purified and checked for quality and final concentration using an Agilent 2200 Tapestation with a High Sensitivity DNA chip (Agilent Technologies, Cary, NC) and a Qubit fluorometer (Thermo Fisher, Raleigh, NC). The final quantified libraries were pooled in equimolar amounts for clustering and sequencing on an Illumina HiSeq 2500 DNA Sequencer, utilizing a 125 bp single end sequencing reagent kit (Illumina, USA). The software package Real Time Analysis (RTA), was used to generate raw bcl, or base call files, which were then de-multiplexed by sample into fastq files for data submission. Data analysis was performed in consultation with Bioinformatics Core at NCSU Center for Human Health and the Environment. We have generated on average ~36 million single-end raw RNAseq data for each replicate. The quality of sequenced data was assessed using FastQC and 12 poor quality bases were trimmed from the 5’-end. The remaining good quality reads were aligned to the zebrafish reference genome (danRer10 version 87) using STAR aligner.^2^ For each replicate, per-gene counts of uniquely mapped reads were calculated using *htseq-count* script from the HTSeq python package.^3^ We imported the count matrix to R statistical computing environment for further analysis. Initially, genes that had no count in most of the replicate sample were discarded. The remaining count data was normalized for sequencing depth and distortion, and dispersion was estimated using DESeq2 Bioconductor package in the R statistical computing environment.^4^ We fitted a leaner model using the treatment levels, and differentially expressed genes were identified after applying multiple testing corrections using the Benjamini-Hjockberg procedure (padj<0.05).^5^ Ingenuity Pathway Analysis (IPA) version 01-10(01-10) provided predictive models of pathway modulation based on transcript abundance (VDD vs. VD3 sufficient).^6^ Using the ‘Pathways and Tox Lists’ tool, “Growth Hormone Siganling” and “IGF-1 Signaling” pathways were loaded separately into the ‘Canonical Pathways’ window. Within the ‘Canonical Pathways’ window, overlay and molecule activity predictor (MAP) functions were applied to predict upstream and downstream effects of our dataset on in silico changes to each known pathway. All significant genes found within our dataset were then loaded into R statistical software and stratified based on fold change (FC) as upregulated (FC>0) and downregulated genes (FC< 0). The KEGG pathway enrichment analysis was conducted using gage (release 3.11) and pathview R package.^7,8,9^ Significant KEGG pathways were identified for based category if p-value < 0.05. Next, gene set enrichment analysis (GSEA (version 4.0.3)) software (Subramanian et al., 2005)⁠ was used to show statistically significant differences between VitD- (VDD) and VitD+ (VD3 Sufficient) datasets.^10^ Briefly, we pre-ranked all genes that were measured using RNA-Seq using *fold change * -log10(p value)* and sorted in descending order. For the annotation mapping, we downloaded an annotation that mapped Zebrafish Ensembl Gene ID to human gene symbol using Ensembl BioMart and used as Chip platform to remap Zebrafish to human orthologues. Enrichment analyses were conducted against curated C2 all version 7.1 symbols. In the GSEA application we modified the following parameters (Enrichment statistics: classic, exclude large sets: 5000, exclude smaller sets: 15). We then assessed the top 20 differentially enriched gene sets and chose to present the most relevant gene set based on our described phenotypes.

**References**

1. Yang, B.-Y. *et al.* Different physiological roles of insulin receptors in mediating nutrient metabolism in zebrafish. *Am. J. Physiol.-Endocrinol. Metab.* (2017) doi:10.1152/ajpendo.00227.2017.

2. Dobin, A. *et al.* STAR: ultrafast universal RNA-seq aligner. *Bioinforma. Oxf. Engl.* **29**, 15–21 (2013).

3. Anders, S., Pyl, P. T. & Huber, W. HTSeq--a Python framework to work with high-throughput sequencing data. *Bioinforma. Oxf. Engl.* **31**, 166–169 (2015).

4. Love, M. I., Huber, W. & Anders, S. Moderated estimation of fold change and dispersion for RNA-seq data with DESeq2. *Genome Biol.* **15**, 550 (2014).

5. Acharya, A. A Complete Review of Controlling the FDR in a Multiple Comparison Problem Framework -- The Benjamini-Hochberg Algorithm. *ArXiv14067117 Stat* (2014).

6. Krämer, A., Green, J., Pollard, J. & Tugendreich, S. Causal analysis approaches in Ingenuity Pathway Analysis. *Bioinformatics* **30**, 523–530 (2014).

7. Kanehisa, M. & Goto, S. KEGG: Kyoto Encyclopedia of Genes and Genomes. 4.

8. Kanehisa, M., Sato, Y., Furumichi, M., Morishima, K. & Tanabe, M. New approach for understanding genome variations in KEGG. *Nucleic Acids Res.* **47**, D590–D595 (2019).

9. Kanehisa, M. Toward understanding the origin and evolution of cellular organisms. *Protein Sci.* **28**, 1947–1951 (2019).

10. Subramanian, A. *et al.* Gene set enrichment analysis: A knowledge-based approach for interpreting genome-wide expression profiles. *Proc. Natl. Acad. Sci.* **102**, 15545–15550 (2005).

**Figures**

**S Table 1**. RT-qPCR primer sequences.

| **Gene** | **Primer Sequence (5’- 3’)** | **Product Size (bp)** |
| --- | --- | --- |
| **Housekeeping** | | |
| *ef1a* | TACAAATGCGGTGGAATCGAC (forward)  GTCAGCCTGAGAAGTACCAGT (reverse) | 246 |
| **Vitamin D Metabolism** | | |
| *cyp2r1* | AGAGGATGCCGTATGTGGAG (forward)  TGCAGTCTAGAAAGCGCTCT (reverse) | 219 |
| *cyp24a1* | ACTCACCAACAACGTGCATC (forward)  CCGCAGTCTTCTTCTTCAGC (reverse) | 234 |
| *cyp27b1* | TCTATCCTGTTATTCCAGCCAA (forward)  GCCTGAAGGAGTCTGGATCT (reverse) | 150 |
| **Lipid Transport** | | |
| *apoBa* | TGACCTCAAGCACGTCACTC (forward)  GGGGAAAACCAGCACTTGTA (reverse) | 167 |
| *apoBb.1* | GCTTGAAGGAACCAGCAGTC (forward)  AGTTGGTGGTTGGCATTAGC (reverse) | 198 |
| *apoA-1b* | GCCCTACGTCCAGGAGTACA (forward)  TTACTCCTTGCTGGCGAACT (reverse) | 169 |
| *apoA2* | AGTTGAGCCAGCACTAGAGG (forward)  ACGTTGTTGATGGCACTGTC (reverse) | 225 |
| *apoC2* | CTACGCCGGCATCTTTCAAG (forward)  TGAAGACAAGAGTTCGCCAC (reverse) | 203 |
| *apoC4* | ACACTGAACCGGATCCCAAA (forward)  GGCCCAATCCATGTAAGAGT (reverse) | 339 |
| *apoEb* | ATTAGCGAGCTGATGAAGGC (forward)  TGTGGCCTCCTTGATCTTGT (reverse) | 213 |
| *fabp11a* | GAGAGAGAGGTGTCGGATGG (forward)  TCAGTGAGCATTTGCTTTGG (reverse) | 224 |
| **Mitochondrial Function** | | |
| *ppargc1a* | CTGCCTTGGTTGGTGAAGAC (forward)  CCAGCAAGTTGGCCTCATTT (reverse) | 210 |
| **Lipogenic Factors** | | |
| *cebpa* | TTAAAGGAGATCCCGTGTGG (forward)  CCTCGTAGAGGTTTGCTTGC (reverse) | 212 |
| *pparaa* | TCCACATGAACAAAGCCAAA (forward)  AGCGTACTGGCAGAAAAGGA (reverse) | 203 |
| *pparg* | CTCTGGAACTGGAGGAGCTG (forward)  TGCTGTGTTCTGGCCTGTAG (reverse) | 216 |
| *srebf1* | ACTAACCGACAGCCAAGTGA (forward)  TTGAGAAGGGCAGTGTCGAT (reverse) | 240 |
| **Lipolytic Factors** | | |
| *lipea* | CGGGCATTACTTTACCTGGC (forward)  CGATGCCTAAACCAGTCTGC (reverse) | 250 |
| *lipeb* | TGGCTCCAGACAACATGCTA (forward)  GCAGACGTTAGCAGCTTCTC (reverse) | 209 |
| *erk1* | TGTGATTTTGGGTTGGCGAG (forward)  GGTCCAGATAGTGCTTCCCA (reverse) | 211 |
| *erk2* | GTTGAAGACGCAGCACTTGA (forward)  ACAGGTTTGATGGCTTCAGG (reverse) | 122 |
| **Growth Hormone Signaling** | | |
| *ghra* | CATTGTCATCTCCCAGCAGC (forward)  TTGTCTGCAGGATCGTCGAT (reverse) | 216 |
| *ghrb* | TATGAACTCAGAGTCCGGGC (forward)  GGAACGGGTGGCAAGAAAAT (reverse) | 206 |
| *ghrh* | TGGAAGACATGCTGATGCCA (forward)  TCCACATCTTGCTTGTAGGTGT (reverse) | 171 |
| *gh1* | TCGTTCTGCAACTCTGACTCC (forward)  CCGATGGTCAGGCTGTTTGA (reverse) | 161 |
| **IGF Signaling** | | |
| *igf1* | GAGTACCCACACCCTCTCAC (forward)  TGAAAGCAGCATTCGTCCAC (reverse) | 213 |
| *igf2a* | GAGGAATGCTGCTTTCGGAG (forward)  GCAGAATGGATGGGACTCCT (reverse) | 217 |
| *igf2b* | CCTGTCTGCCTTCGAAGTTG (forward)  AACGTCCCTCTCTGACTTGG (reverse) | 232 |
| *igf1ra* | TTCTCCTGTTCCGTGTCTCC (forward)  CCGAATCCAAGTAGCACAGC (reverse) | 210 |
| *igf1rb* | AGCCTTCGAGAACTTCCTCC (forward)  AAACGGTAAAAGGCTGCAGG (reverse) | 239 |
| *igfals* | GAGACAGGCTTTCAGGACCT (forward)  AATGCTCTAGGTGCCACAGT (reverse) | 227 |
| **Insulin Signaling** | | |
| *insa* | AGTGTAAGCACTAACCCAGGCACA (forward)  TGCAAAGTCAGCCACCTCAGTTTC (reverse) | 177 |
| *insb* | CTCTGCTCACTCAGGAAAAGG (forward)  GGATGGAGAAGACTGCGAT (reverse) | 124 |
| *insra* | GTGTCGAAAAGTGCCCACAT (forward)  TCAACCCCATGCACACTTTG (reverse) | 237 |
| *insrb* | GACTGATTACTATCGCAAGGG (forward)  TCCAGGTATCCTCCGTCCAT (reverse) | 210 |
| *irs1* | ACAACAAGTACGCCTCCAGA (forward)  GCTGGGAATTGCTTTGACCA (reverse) | 220 |
| *irs2a* | CCGGTCTTTCCCTCTTTTGC (forward)  TCCTGCTTTACAACAACCGC (reverse) | 240 |
| *irs2b* | CCAGTGTTTCCCTCCTTTGC (forward)  CTAGCAGCTTTGAACCGGAC (reverse) | 227 |
| *insig1* | TGCTGGACACGCTATTTTCG (forward)  TTGATGCCCACAAAGACAGC (reverse) | 172 |
| *insig2* | CAGACGTCATCAGCAGCATC (forward)  TCAGGGACAGCTGCACATTA (reverse) | 228 |
| *cidec* | ATCAGCCTGTGGTTCTGTGA (forward)  GTGGTGCAGGTCTTCTAGGT (reverse) | 133 |
| **Suppressors of Cytokine Signaling** | | |
| *cish* | ACACCAGCAGCACAGAAATG (forward)  AGAGACTTGCTGACCGTCAA (reverse) | 231 |
| *socs1a* | ACAAAGCACAAAACGGTCCC (forward)  GGATTGGTTGCACAGGGATG (reverse) | 249 |
| *socs1b* | ACAACGGCCATACAAATGCA (forward)  AAGAAAACGTTGGCCTGCAT (reverse) | 247 |
| *socs2* | GGCAAGTTCAAGCTGGACTC (forward)  ACCCGTCGTGTGGTTTTATTG (reverse) | 239 |
| *socs3a* | CTCCAAGAGCATGCAATCCG (forward)  TGTCCGTTCACAGTCTTCCT (reverse) | 210 |
| *socs3b* | AGACACTCGGTTTCCTCCAG (forward)  CAATGCGTTCGCTCTAGTCC (reverse) | 216 |
| *socs6a* | GCCATCATTACAGCCCTTCG (forward)  GTCCTGAGGTGTGAGTCCAA (reverse) | 248 |
| *socs6b* | ATTTGCTGGTACACACGCTC (forward)  TTCTTCAGCCTCCCATCTGG (reverse) | 205 |
| *stat3* | AAACTACTCCGGTTGCCAGA (forward)  ATTCCTCCCTCTTTGCTGCT (reverse) | 246 |
| *stat5b* | TTATCCCTGCCTGTTGTGGT (forward)  TGAGCGAGGAACACCAGATT (reverse) | 221 |

**S Table 1**. Full list of RT-qPCR primer sequences. Bold titles indicate gene category. Gene expression was normalized to *efla* as the housekeeping gene.

**S Table 2.** Whole fish bone density analysis using microCT.

| Sample Name | Total Volume (mm^3) | Bone Volume (mm^3) | BV/TV | Whole Fish Density (HU) | Bone Density (HU) | Bone Surface Area (mm^2) | Bone BS/BV |
| --- | --- | --- | --- | --- | --- | --- | --- |
| VD3 Sufficient (mean) | 209.00 | 7.12 | 0.03 | -30.15 | 560.18 | 317.56 | 46.10 |
| Sufficient (SD) | 50.17 | 2.70 | 0.01 | 2.55 | 14.13 | 93.66 | 4.68 |
| VDD (mean) | 78.33 | 2.01 | 0.03 | -41.39 | 427.98 | 107.11 | 55.76 |
| Deficient (SD) | 5.50 | 0.43 | 0.01 | 3.04 | 27.12 | 16.34 | 4.41 |

**S Table 2.** VDD fish had less total volume (78.33±5.50mm^3^), bone volume (2.01±0.43mm^3^), whole fish density (-41.39±3.04HU), bone density (427.08±27.12HU), and bone surface area (mm^2^) 6 mpf.

**S Table 3**. Whole fish calcium levels using ICP MS.

| Sample Name | Avg. (ug) Calcium per Whole Fish | Avg. Calcium per Whole Fish Normalized to Body Weight (ug/g) |
| --- | --- | --- |
| VD3 Sufficient | 1256.22 ± 225.25 | 4656.1194 ± 499.50 |
| VDD | 193.90 ± 56.46 | 3060.2439 ± 442.05 |

**S Table 3**. VDD fish had less whole fish calcium (193.90±56.46ug; 3060.2439±442.05ug/g) 6 mpf.

**Figure Captions**

**S Figure 1.** Stunted growth observed in the VDD zebrafish 6 mpf. (A) Both male and female VDD zebrafish exhibited decreased body weight 6 mpf. Body weight was taken biweekly starting at 2 mpf and ending at 6 mpf (2 mpf, 2.5 mpf, 3 mpf, etc.). At 6 mpf, the average body weight (g) for VDD, VD3 sufficient, and LD fish was 0.0525±-0.007g, 0.1761±0.015g, and 0.2403±0.0230g, respectively. Data are represented as mean±SEM. (B) The BWI (g) from 2-6 mpf for VDD, VD3 sufficient, and LD fish was 0.0464g, 0.1700g, and 0.2342g, respectively. (C) Feeding rate was taken every month starting at 2 mpf and ending at 6 mpf (2 mpf, 3 mpf, 4 mpf, etc.). The feeding rate (%) at 6 mpf for VDD, VD3 sufficient, and LD fish was 4.11±0.0006%, 4.68±0.0030%, and 1.62±0.0020%, respectively. Data are represented as mean±SEM. All measures are representative of a mixed gender population.

**S Figure 2.** Determining if vitamin D supplementation can promote growth and fat loss. (A-B) SL (cm) and weight (g) were taken biweekly starting at 2 mpf and ending at 11 mpf. At 6.5pmf, after 2 weeks on the vitamin D supplemented diet, VDD + VD3 fish exhibited greater SL and weight (1.56±0.06cm; 0.07±0.01g) than VDD fish. (C) Descending order: MALE (lab diet, VD3 sufficient, VDD + VD3, VDD).

**S Figure 3.** Determining if VDD promotes glycogen storage in the liver. (A) VD3 sufficient liver H&E. (B) VDD liver H&E. (C-D) VD3 sufficient liver before (C) and after (D) PAS digestion. (E-F) VDD liver before (E) and after (F) PAS digestion.

**S Figure 4**. RNA-Seq Ingenuity Pathway Analysis (IPA) (version 01-10(01-10)) showing predicted activation or deactivation of genes based on transcript abundance in VDD liver compared to VD3 sufficient liver 6 mpf. (A) GH signaling cascade. (B) IGF signaling cascade. The signaling cascades were generated through the use of IPA (QIAGEN Inc., https://www.qiagenbio- informatics.com/products/ingenuity-pathway-analysis).^6^

**S Figure 5**. RNA-Seq KEGG (version 3.11) analysis of INS signaling pathway showing predicted activation or deactivation of genes based on transcript abundance in VDD liver compared to VD3 sufficient liver 6 mpf.^6,7,8^ <https://bioconductor.org/packages/release/bioc/html/gage.html>

**S Figure 6**. GSEA (version 4.0.3) enrichment analysis of RNA-Seq dataset based on transcript abundance in VDD liver compared to VD3 sufficient liver 6 mpf. (A) Bar plot of top 20 differently enriched gene sets with the metabolic syndrome gene set outlined in red. (B) Enrichment analysis for metabolic syndrome gene network. <https://www.gsea-msigdb.org/gsea/index.jsp>
