# Supplementary figures and images for: Vitamin D deficiency serves as a precursor to stunted growth and central adiposity in zebrafish
Source: Sci Rep. 2020 Sep 29;10:16032. doi: 10.1038/s41598-020-72622-2 (PMC7524799; doi:10.1038/s41598-020-72622-2)

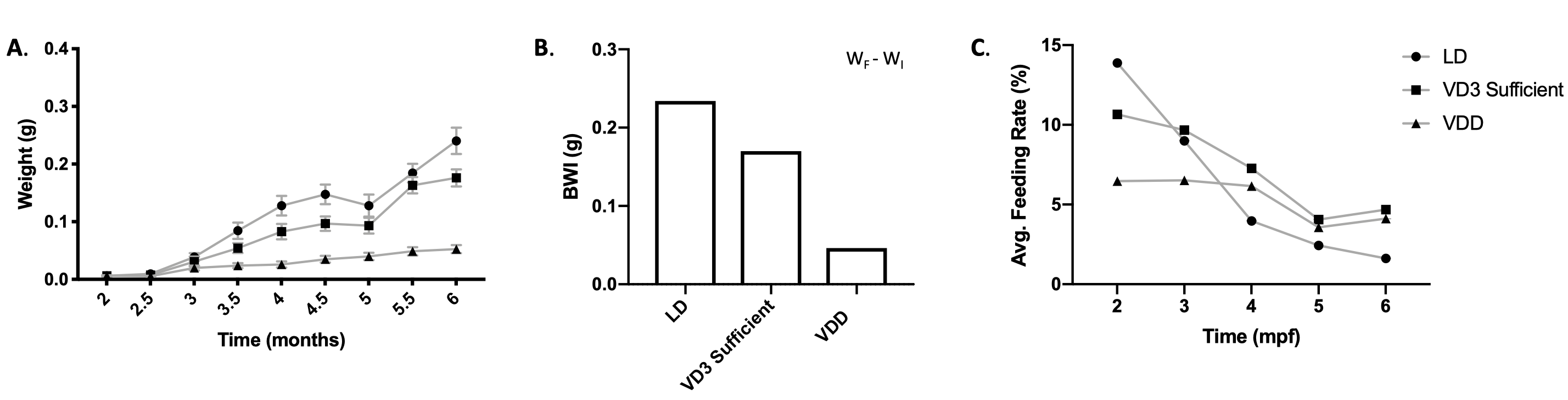

Supplement: Supplementary file 2 — Supplementary Figure 1. [file 41598_2020_72622_MOESM2_ESM.tiff]

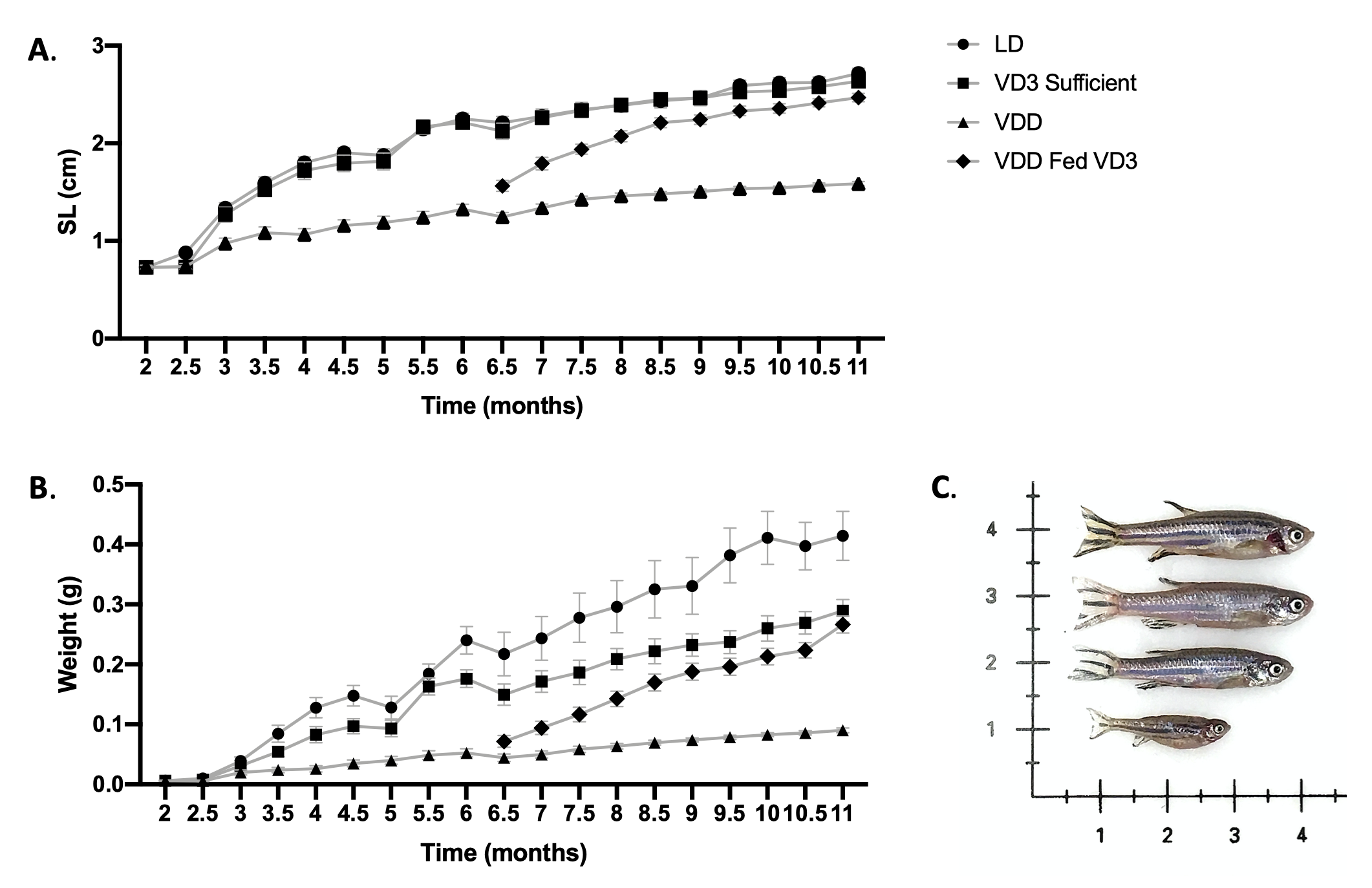

Supplement: Supplementary file 3 — Supplementary Figure 2. [file 41598_2020_72622_MOESM3_ESM.tiff]

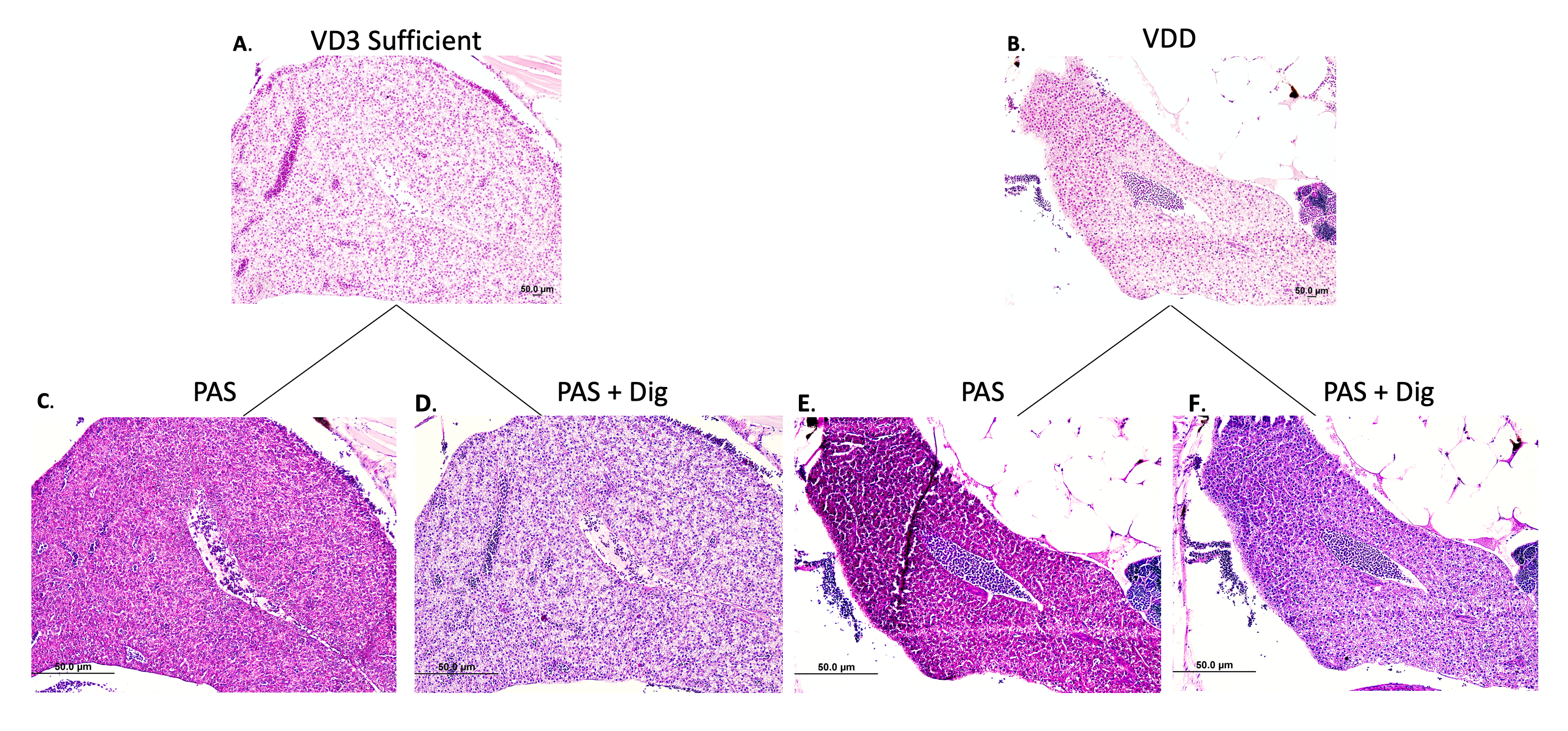

Supplement: Supplementary file 4 — Supplementary Figure 3. [file 41598_2020_72622_MOESM4_ESM.tiff]

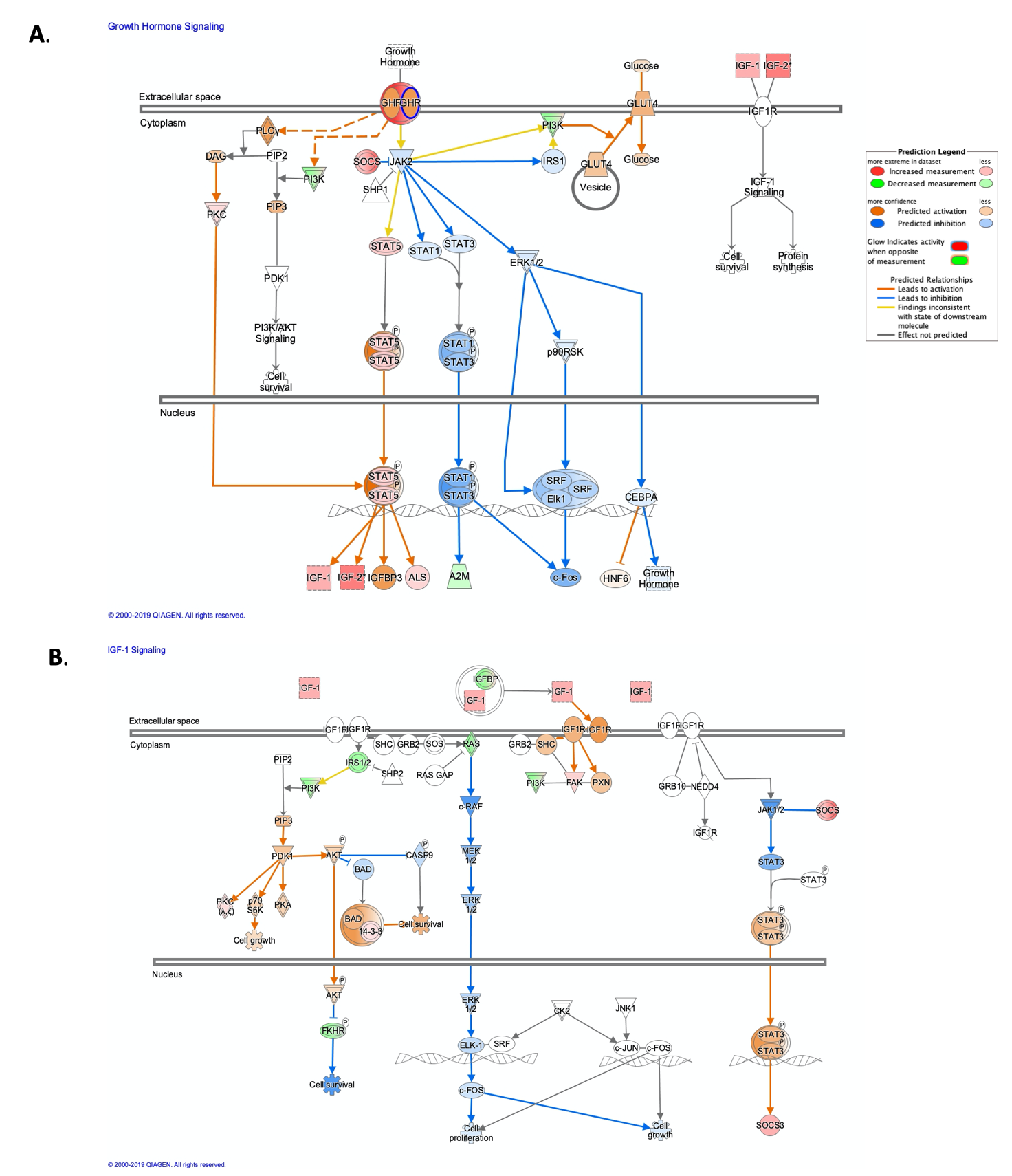

Supplement: Supplementary file 5 — Supplementary Figure 4. [file 41598_2020_72622_MOESM5_ESM.tiff]

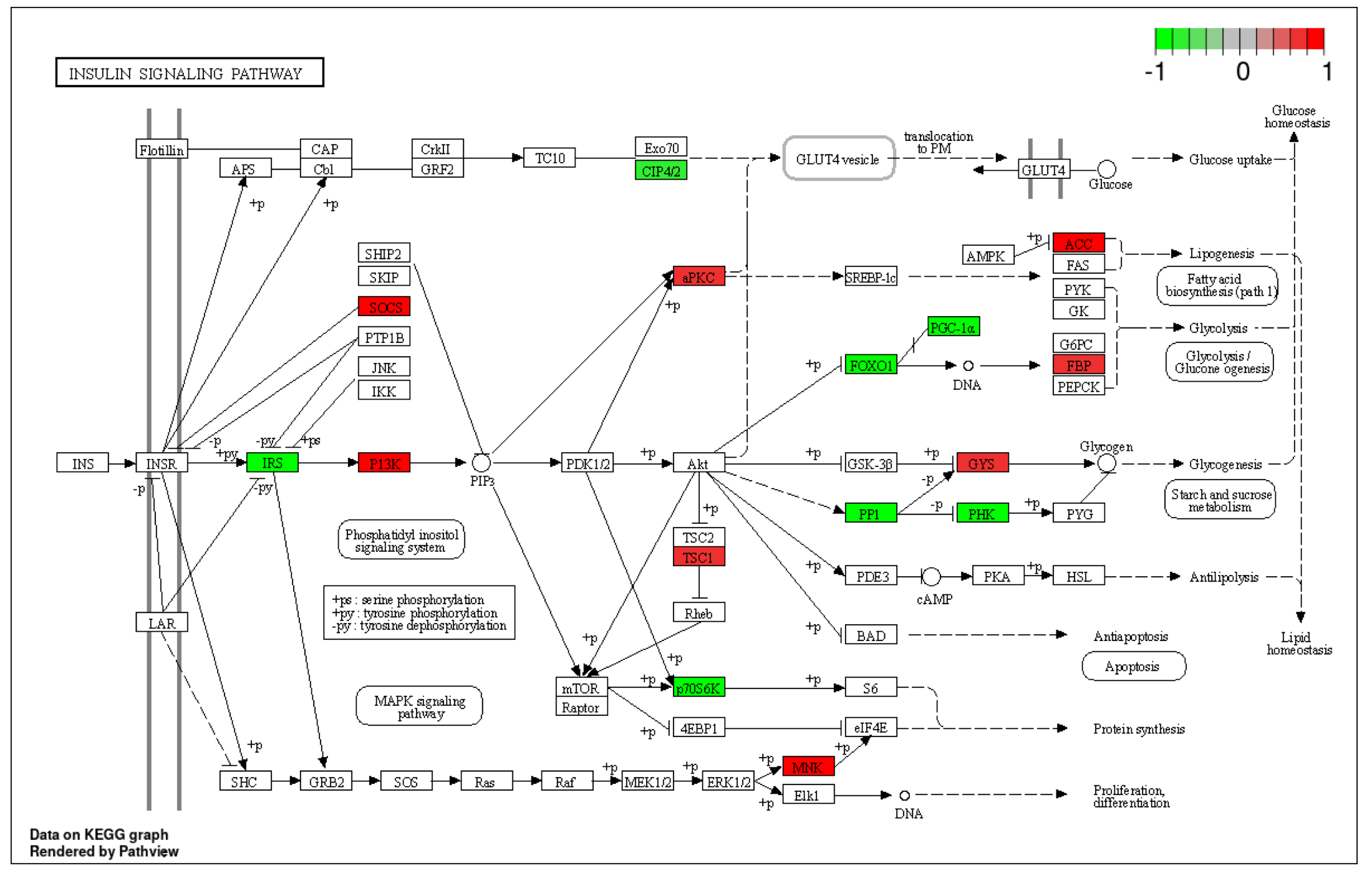

Supplement: Supplementary file 6 — Supplementary Figure 5. [file 41598_2020_72622_MOESM6_ESM.tiff]

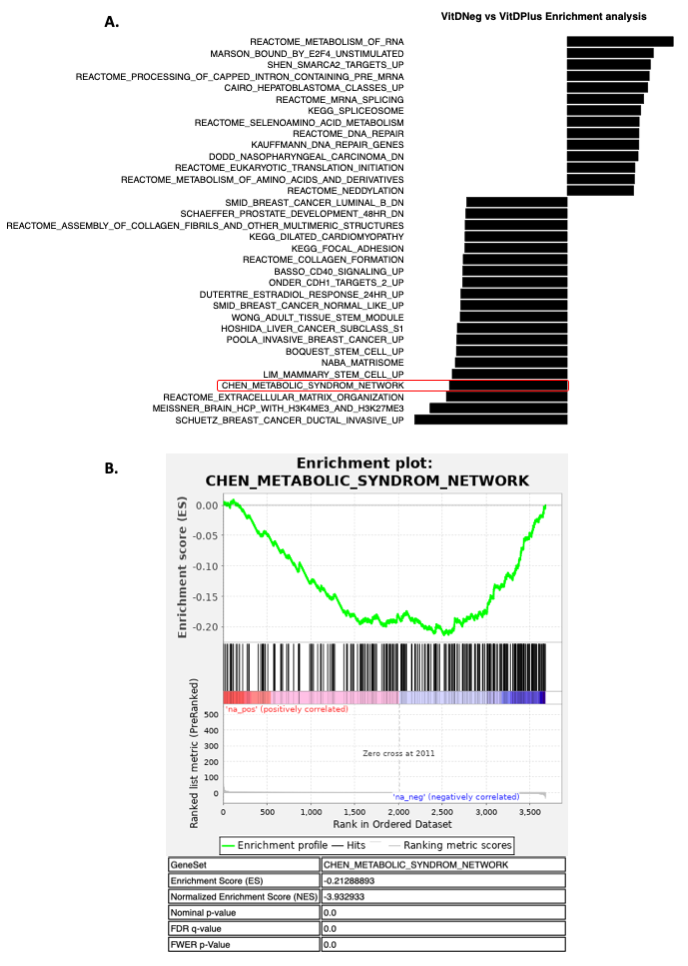

Supplement: Supplementary file 7 — Supplementary Figure 6. [file 41598_2020_72622_MOESM7_ESM.tiff]
